# A principal components method constrained by elementary flux modes: analysis of flux data sets

**DOI:** 10.1186/s12859-016-1063-0

**Published:** 2016-05-04

**Authors:** Moritz von Stosch, Cristiana Rodrigues de Azevedo, Mauro Luis, Sebastiao Feyo de Azevedo, Rui Oliveira

**Affiliations:** REQUIMTE/DQ, Faculty of Science and Technology, University Nova de Lisboa, Campus de Caparica, 2829-516 Caparica, Portugal; DEQ, Faculty of Engineering, University do Porto, Rua Dr. Roberto Frias s/n, 4200-465 Porto, Portugal

**Keywords:** Flux data analysis, Fluxome data analysis, Principal component analysis, Elementary flux modes, Principle elementary modes

## Abstract

**Background:**

Non-negative linear combinations of elementary flux modes (EMs) describe all feasible reaction flux distributions for a given metabolic network under the quasi steady state assumption. However, only a small subset of EMs contribute to the physiological state of a given cell.

**Results:**

In this paper, a method is proposed that identifies the subset of EMs that best explain the physiological state captured in reaction flux data, referred to as principal EMs (PEMs), given a pre-specified universe of EM candidates. The method avoids the evaluation of all possible combinations of EMs by using a branch and bound approach which is computationally very efficient. The performance of the method is assessed using simulated and experimental data of *Pichia pastoris* and experimental fluxome data of *Saccharomyces cerevisiae*. The proposed method is benchmarked against principal component analysis (PCA), commonly used to study the structure of metabolic flux data sets.

**Conclusions:**

The overall results show that the proposed method is computationally very effective in identifying the subset of PEMs within a large set of EM candidates (cases with ~100 and ~1000 EMs were studied). In contrast to the principal components in PCA, the identified PEMs have a biological meaning enabling identification of the key active pathways in a cell as well as the conditions under which the pathways are activated. This method clearly outperforms PCA in the interpretability of flux data providing additional insights into the underlying regulatory mechanisms.

**Electronic supplementary material:**

The online version of this article (doi:10.1186/s12859-016-1063-0) contains supplementary material, which is available to authorized users.

## Background

An elementary flux mode (EM) represents a unique and non-decomposable sub network of metabolic reactions that works coherently in steady state [1]. Elementary flux mode analysis has proven to be a powerful method to understand the structural properties of metabolic networks [1–5]. For example, this approach can be employed to assess which reactions and educts are involved in producing a certain compound, to determine optimal yields or to analyze the consequences of certain reactions taking a zero value as invoked by metabolic engineering or changes in the cellular environment [6].

The material balances of a metabolic network in steady state take the form of a system of linear algebraic equations:

1 $$ 0=S\cdot v $$

with *S* the metabolic network stoichiometric matrix (dim(*S*) = *n*_*c*_ × *n*_*v*_) and *v* a vector of reaction fluxes (dim(*v*) = *n*_*v*_). A set of *i* = 1, …, *n*_*d*_ flux distributions *V* = {v_*i*_}(dim(*V*) = *n*_*v*_ × *n*_*d*_) obeying to eq. (1) can be further expressed as a non-negative linear combination of EMs:

2 $$ V=E\cdot P={\displaystyle {\sum}_{i=1}^m{e}_i\cdot {p}_i} $$

with *E* a matrix of *m* EMs and *P* a matrix of weighting factors that quantify the contributions of the EMs to the observed fluxes *V* [1]. Nonzero values in *p*_*i*_ indicate how the *e*_*i*_ contribute to flux-phenotype [7, 8]. Investigating which of the *p*’s have nonzero contributions for a given phenotype is useful for two reasons [8]: 1) The biological interpretability of EM-based pathway analysis is improved, which can help to focus on studying physiologically active processes; and 2) Changes in the physiological state of the cell can be quantified, enabling the causes of change to be elucidated.

Different methods have been proposed to analyze those *p*’s which have nonzero contributions. Ferreira et al. [7] outlined that different principles, such as network connectivity and stoichiometry [9, 10], thermodynamics [11, 12], or enzyme kinetics [8], can be used to identify EMs that cannot be active. Though these approaches are good to reduce the number of EMs beforehand, they do not provide specific values for *p*’s, the contributions from the remaining EMs.

Several methods have been proposed that combine nonlinear programming and experimental data [13–16]. Palsson and co-authors [15, 16] suggested a method for maximizing and minimizing the contributions of extreme pathways (the systemically independent subset of EMs) for a given steady-state flux distribution using linear optimization. This yields ranges of possible non-negative contribution values associated with the extreme pathways, the so called alpha spectrum. The alpha spectrum typically indicates that many extreme pathways could be active simultaneously. However, several studies suggest that the regulation problem is of low dimensionality [17–21], wherefore only a reduced set of extreme pathways or EMs can be expected to be active. Wang et al. [14] proposed to randomly sample *u* EMs several times, each time minimizing a least square functional consisting of the flux data and a flux distribution simulated with *u* EMs, in order to identify which set of *u* active EMs explains the flux data best. Different values of *u* have to be tested to identify how many EMs are active. Since the number of theoretically possible combinations of EMs grows with increasing the number of EMs and almost exponentially with increasing values of *u* (${n}_{comb}=\frac{m!}{u!\cdot \left(m-u\right)!}\approx \frac{m^u}{u!}$), the number of times the random sampling has to be executed also grows with increases in both *m* and *u*. Nookaew et al. [13] suggested to use mixed integer linear programming to determine the active EMs and their contributions from yield data. However, also for this algorithm the number of EM combinations that have to be evaluated increases with the number of EMs and active EMs.

Ferreira et al. [7] proposed a method that maximizes the variance between data of the extracellular environment and data of the reaction fluxes. While this approach allows inferring which EMs are active under certain environmental conditions, the identification of the nonzero *p* contributions is dependent on the appearance of evidence in the environmental data that indicates changes in the *p* contributions. Barret et al. [17] performed a basis rotation on the loadings obtained from principal component analysis of flux data to find the “eigenfluxes” – sets of independently-operable reactions, which allow for a biological interpretation of the principal components. However, different basis rotation approaches can yield different eigenfluxes for the same loadings [17].

In this study, the aim is to infer the nonzero *p* contributions directly from reaction flux data. The decomposition of the flux distributions into EMs is not straightforward [8]. It is for instance not possible to regress the flux matrix with all EMs, since the number of EMs is typically much greater than the number of experiments in which fluxes were measured, such that the system of linear equations, eq. (2), is underdetermined. In addition, the EMs are typically not orthogonal to one another so that the summation of contributions obtained when regressing two EMs, one at a time, yields a different result than when regressing both simultaneously (see Additional file 1). In what follows, a methodology is proposed, which identifies the combination of EMs that best captures the patterns observed in reaction flux data, i.e. the principal EMs (PEMs), given a specific number of PEMs.

## Methods

The difficulty in interpretation of Principal Component Analysis (PCA) [22] data was the main motivation for the development of the Principal Elementary Mode Analysis (PEMA) method proposed here. In PCA a matrix of data, *X*, is decomposed into matrices of loadings *W* and scores *T* such that a maximum amount of variance of the data is captured in an underlying latent space for a specified number of latent variables, *n*_*lv*_:

3 $$ X=W\cdot T={\displaystyle {\sum}_{n_{lv}}{w}_{n_{lv}}\cdot {t}_{n_{lv}}}. $$

The scores describe the patterns in the data in the underlying (orthogonal) latent space and the loadings describe the relationship between the latent space and the patterns in the data. The PCA loadings are determined in an iterative procedure from the data, *X*, such that for each latent variable a maximum of variance in the data can be captured [22].

The structural resemblance between the EM equation (2) and the PCA equation (3) is obvious. However, in PCA the principal components (the loadings) are loose structures determined by measured flux data in the sense of variance maximization. In contrast, PEMA is constrained by all possible loading combinations, i.e. the complete (large) set of EMs, which are fixed a priori and determined by the metabolic network structure. Thus the challenge in PEMA is to identify the minimal subset of “active” EMs, i.e. the principal EMs (PEMs), that maximize the explained variance of flux data, *V*_*mes*_.

### The Principal Elementary Mode Analysis (PEMA) method

The PEMA algorithm consists of three steps. At first the available EMs are analyzed by comparing with one another and with respect to the available flux data, which allows reducing the number of feasible EM combinations. In the second step, a greedy approach is used to determine a best first combination, which serves as a lower bound in the following, step 3, the branch and bound EM selection method.

#### Step 1: Pre-selection and analysis of the EMs

Each *e*_*i*_ has size dim(*e*_*i*_) = *n*_*v*_ fluxes. However, the number of measured fluxes (*n*_*v*,*mes*_) is typically much lower than *n*_*v*_, comprising only a subset of fluxes. This has direct consequences for the identification of the active EMs, since it will not be possible to distinguish between EMs that have zero or equal contributions in the EM entries of the measured fluxes, termed hereafter ambiguous EMs. Thus, at this stage, the EMs are filtered with respect to this ambiguity (only one of the ambiguous EMs is kept in the set), but the information about which EMs are ambiguous is saved for analysis of the selected EMs, in case that an ambiguous EM is selected by the algorithm. (Note, which and how many of the EMs are ambiguous depends on the specified measured fluxes *V*_*mes*_ and the metabolic network. The ambiguity of the EMs is directly related to the question whether the system *V*_*est*_ = − *S*_*est*_^#^ ⋅ *S*_*mes*_ ⋅ *V*_*mes*_ is 1) determined (no ambiguous EMs) or 2) underdetermined (ambiguous EMs) for the specified measured fluxes, with the set of flux distributions $$ V=\left[\begin{array}{c}\hfill {V}_{mes}\hfill \\ {}\hfill {V}_{est}\hfill \end{array}\right] $$ and *V*_*est*_ the matrix of unmeasured fluxes).

In the next pre-selection step the directions of the EM contributions are analyzed. Due to the non-cancelation principle [6, 23], i.e. a reaction can only be active in one direction at one time, the flux contributions of the EMs that can be chosen must obey the direction imposed by the measured flux data *V*_*mes*_. Thus, possible errors in the direction of the measured flux data must be addressed before filtering the EMs.

#### Step 2: A “best first” EM combination by means of a greedy approach

A “best first” combination is obtained employing a greedy approach that iteratively decomposes the flux patterns of *V*_*mes*_ identifying which EM contributes the most until the given number of EMs that should be combined (*n*_*Fac*_) is reached. In the beginning each EM *i* of all *m* EMs is divided by its 2-norm value, i.e.:

4 $$ {e}_{i,n}=\frac{e_i}{{\left\Vert {e}_i\right\Vert}_2}\forall\ i=1..m $$

where *e*_*i*,*n*_ is the norm-scaled *i*^th^ EM and *m* the number of EMs. This scaling makes the following manipulations easier and it does not change the ratio between the elements of each EM vector, but it only scales the weights of the *i*^th^ EM by its norm: *p*_*i*,*n*_ = *p*_*i*_ ⋅ ‖*e*_*i*_‖_2_. In each iteration, the vectors of scaled weights, *p*_*i*_, are determined by regressing the flux matrix *V*_*iter*_ with the respective EM, which for the *i*^th^ EM gives:

5 $$ {p}_{i,n}={e_{i,n}}^T\cdot {V}_{iter}, $$

because *e*_*i*,*n*_^*T*^ ⋅ *e*_*i*,*n*_ = 1. Some of the values in the vectors of weights, or even all, might be negative, which is in conflict with the definition that the weights need to be greater or equal than zero [1]. In order to account for this constraint the negative values in the vectors of weights are replaced by zeros. The consequence of replacing negative weight values with zeros, is that the variance related to the negative values is not extracted from *V*_*iter*_. In the next step *p*_*i*,*n*_ is used to calculate the contribution of the *i*^th^ EM to the fluxes, *V*_*est*,*i*_:

6 $$ {V}_{est,i}={e}_{i,n}\cdot {p}_{i,n}. $$

The flux contributions for each of the EMs are then compared to the measured flux values using the explained variance criteria:

7 $$ {\vartheta}_i=1-\frac{{\displaystyle {\sum}_k}{\displaystyle {\sum}_j}{\left({V}_{mes,j,k}-{V}_{est,i,j,k}\right)}^2}{{\displaystyle {\sum}_k}{\displaystyle {\sum}_j{V_{mes,j,k}}^2}}, $$

Where *k* sums over the number of data points, *j* sums over the number of fluxes and *V*_*mes*,*j*_ is the measured flux *j*. It is shown in the Additional file 1 that this equation can be simplified to:

8 $$ {\vartheta}_i=\frac{{\displaystyle {\sum}_k}{\displaystyle {\sum}_{l=1}^i}{p_{l,n}}^2}{{\displaystyle {\sum}_k}{\displaystyle {\sum}_j{V_{mes,j,k}}^2}}. $$

According to the calculated explained variance values the EMs can be ranked. The EM, which yields the greatest variance value, is selected in each iteration. Then the captured patterns are subtracted from the flux matrix, such that only the unexplained patterns remain:

9 $$ {V}_{iter+1}={V}_{iter}-{V}_{est, selected}, $$

where *V*_*est*,*selected*_ is the flux contribution of the selected EM. Thereupon a new iteration is started. In the first iteration *V*_*iter* = 1_ = *V*_*mes*_. The iterative procedure is stopped if either the number of measured fluxes or the user defined number of EMs that should be combined for explaining the variance in the flux data (referred to as factors, *n*_*Fac*_s) is reached.

#### Step 3: A branch and bound procedure for the identification of the PEMs

**Fig. 1:**
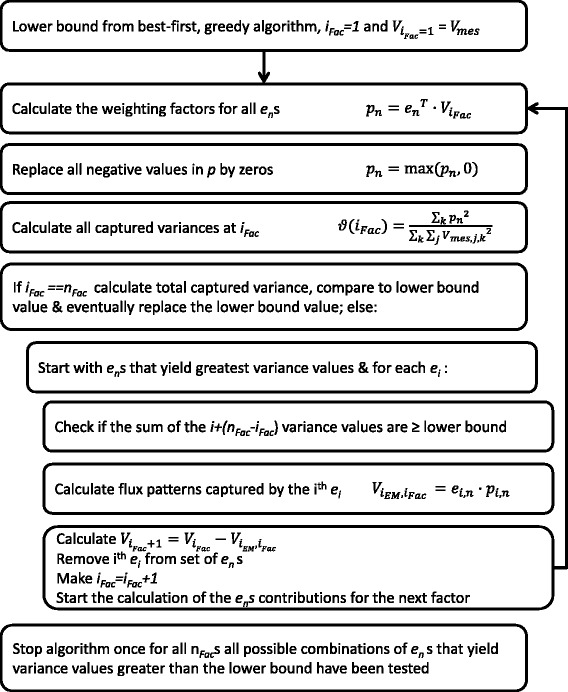
Schematic representation of the branch and bound PEM selection algorithm

In contrast to PCA, the EMs selected by the greedy approach are not necessarily orthogonal to one another, wherefore in contrast to PCA, the EMs selected by the greedy approach cannot be guaranteed to capture the most variance for a given number of factors, i.e.: another combination of EMs with the same number of factors might explain more variance. Therefore, it becomes necessary in principle to exploit all possible combinations of EMs for all factors, which means that a number of combinations ${n}_{comb}=\frac{m!}{n_{Fac}!\cdot \left(m-{n}_{Fac}\right)!}$ need to be evaluated. Thus, the EM selection procedure will have to deal with a combinatorial explosion in the evaluation of possible combinations for an increasing number of factors and EMs. Here, a branch and bound technique is used to reduce the number of evaluations of EM combinations. The steps of the algorithm are visualized in Fig. 1. The procedure starts with calculating the captured variance for each EM *i*, using equations 5, 6 and 8, with *V*_*iter* = 1_ = *V*_*mes*_. For the second factor all possible combinations of EM *i* with each EM *j* of the remaining EMs need to be evaluated. For the following factor all possible combinations of EMs *i*, *j* with the remaining EMs need to be evaluated and so forth until *n*_*Fac*_ is reached. It can be seen that the algorithm performs the combinatorial search by calling itself for each increase in factor and possible elementary mode combinations. However, if the sum of the variances captured at any level for a combination of *n*_*Fac*_ EMs does not reach the lower bound, then this combination is not evaluated, since it cannot capture more variance than had been captured before. The sum of variances calculated for (*n*_*Fac*_ − *i*_*Fac*_) EM combinations at any factor *i*_*Fac*_ is such an upper bound. The upper bound is reached if the (*n*_*Fac*_ − *i*_*Fac*_) EMs are independent, but since this is typically not the case the actual variance is lower. The lower bound is raised every time a combination is encountered that can capture more variance.

A Matlab implementation of the algorithm can be found in the Additional file 2.

## Results and discussion

The proposed method was applied to three case studies, one study with simulation data and two others with experimental data. In the simulation study the flux data were generated using EMs of the metabolic network of *Pichia pastoris*, such that the active EMs are known. Different numbers of EMs are involved in the experimental studies on *Pichia pastoris* and *Saccharomyces cerevisiae*, 98 and 1182 EMs respectively. In all studies the results obtained with the proposed method were compared to results obtained with PCA, which is the standard tool to analyze the latent structure of flux data.

### Pichia pastoris simulation case study

This case is based on the metabolic network of *Pichia pastoris*, which originates from Tortajada et al. [23]. It describes the central carbon metabolism of *P. pastoris* during growth on glucose, glycerol and methanol, comprising the Embden-Meyerhoff-Parnas pathway, citric acid cycle, pentose phosphate and fermentative pathways. It contains 45 compounds (36 of which are internal metabolites, which can be balanced for growth) and 44 reactions, yielding a total number of 98 EMs [23]. Flux data were generated simulating the growth of *Pichia pastoris* for twelve different cultivation conditions by choosing appropriate sets of active EMs (the flux data can be found in the Additional file 3). The active EMs were assumed to contribute randomly to the flux pattern. For more details see the Additional file 1. This allows comparing the set of EMs identified with PEMA to the active EMs that were used for data generation, hereupon termed “active EMs”. This case study also enables the study of the impact of noise on the EMs identification and performance. Only the uptake and secretion flux data were used thus mimicking the experimental study. For each flux the data values, as well as their respective entry in the EMs, were scaled by the mean value of this flux in order to reduce the impact of differences in magnitudes between values of different fluxes.

#### Analysis of the performance without noise

The results obtained with PEMA and PCA are shown in Table 1 in case of no noise added to the data. It can be seen that the number of the selected EMs varies when increasing the number of *n*_*Fac*_s from one to six. For further increases in number of *n*_*Fac*_s, it can be observed that systematically the same (1 to *n*_*Fac*_-1) EMs are selected. From six up to nine *n*_*Fac*_s the EMs selected with PEMA all belong to the set of active EMs. However, the 10^th^ identified EM does not belong to the set of active EMs. It can also be observed that from the 9^th^ EM on the increase of explained variance is negligible (changes are observed only three digits after the decimal point). While more than 97 % of the variance can be explained with the identified first nine EMs, the seven EMs that remain to be identified, generate in total less than 3 % of variance in the 12 simulated experiments. Since there is only little evidence for the activity of these EMs in the data, it will be extremely difficult to identify them, particularly when noise is present in the data. The identified set of EMs therefore is not exclusive.

**Table 1:** Selected EMs and the respective captured variance (ϑ) values for one to 10 number of *n*_*Fac*_s obtained for the simulated data without noise

*n*_*Fac*_	EM/ϑ	1	2	3	4	5	6	7	8	9	10
1	EM	70									
1	ϑ	29.45									
2	EMs	70	7								
2	ϑ	29.45	53.82								
3	EMs	70	7	40							
3	ϑ	29.45	53.82	71.16							
4	EMs	7	69	13	40						
4	ϑ	24.49	46.86	65.83	83.55						
5	EMs	7	71	13	33	37					
5	ϑ	24.49	45.99	64.42	82.48	93.96					
6	EMs	7	33	13	3	37	23				
6	ϑ	24.49	44.02	62.29	78.93	91.37	97.08				
7	EMs	7	33	13	3	37	23	12			
7	ϑ	24.49	44.02	62.29	78.93	91.37	97.08	97.23			
8	EMs	7	33	13	3	37	23	12	19		
8	ϑ	24.49	44.02	62.29	78.93	91.37	97.08	97.23	97.26		
9	EMs	7	33	13	3	37	23	12	19	16	
9	ϑ	24.49	44.02	62.29	78.93	91.37	97.08	97.23	97.26	97.28	
10	EMs	7	33	13	3	37	23	12	19	16	17
10	ϑ	24.49	44.02	62.29	78.93	91.37	97.08	97.23	97.26	97.28	97.28
PCA	n_lv_**	1	2	3	4	5	6	7	8	9	
PCA	ϑ	50.16	82.19	91.85	97.00	99.27	99.96	100.00	100.00	100.00	
BF*	EMs	70	7	40	13	37	23	14	12	8	16
BF*	ϑ	29.45	53.82	71.16	82.44	90.54	91.84	92.04	92.12	92.17	92.20

The set of truly active EMs for data generation was EMs = [1, 3, 7, 12, 13, 14, 16, 19, 20, 22, 23, 24, 28, 32, 33, 37]. BF* best-first identification by the greedy approach. *n*_*lv*_ ** number of latent variables for PCA

When comparing the PEMA final results to the best-first solution (which is used as an initial approximate and lower bound in the first part of the PEMA algorithm), shown in the last rows of Table 1, it is obvious that up to the fifth EM the performance of the best-first solution in terms of captured variance is comparable to the final result, though the first, third and ninth identified EMs do not belong to the set of “active EMs”. The misidentified EMs pose an inductive bias onto the identification of the following active EMs, wherefore the performance for greater number of *n*_*Fac*_s becomes inferior. This was expected as outlined in the method section and the reason why the here proposed PEMA method was developed.

From the results obtained with PCA, also shown in Table 1, it can be concluded that the simulated data can be described on a latent variable space of lower dimension, at maximum nine latent variables are required. While the performance of PCA in terms of explained variance is superior to PEMA, its loadings *W* have no biological meaning, which makes the biological interpretation of the results more difficult.

#### Analysis of the PCA loadings and PEMs

**Fig. 2:**
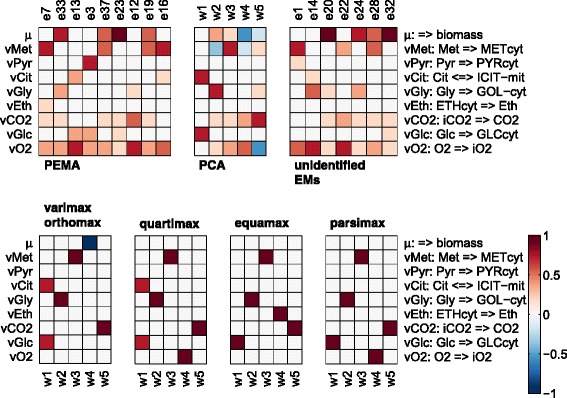
Plot of the active EMs-normalized (on the *upper left* the identified EMs (PEMs), on the *upper right* the unidentified EMs) and the first five PCA loadings, as well as the basis rotated loadings (*lower plots*). The corresponding reactions are shown on the *right*, where => signifies irreversible reactions, whereas <=> reversible reactions

The contributions of the active normalized EMs (segmented into identified (PEMA) and unidentified EMs) to each flux are shown in Fig. 2 together with the contribution of the PCA loadings and basis rotated PCA loadings. The basis rotation of the loadings allows for a more biological interpretation of the principal components [17]. Different orthogonal basis rotation methods were used, namely varimax, orthomax, quartimax, equamax and parsimax. These methods rotate the loadings according to different objectives, for more details see [24, 25]. In case of the identified EMs it can be seen that the EMs contribute to all fluxes. It appears that, in case of PCA, none of the loadings contributes to the ethanol and pyruvate fluxes, but in fact their respective values are only very small. However, the contributions of the PCA loadings to the fluxes are very difficult to interpret. For instance, the negative contributions of *w*_3_ to the methanol flux paired with positive contributions of this loading to the glycerol and the oxygen flux does not make sense from a biological point of view, since this would either mean that i) glycerol and oxygen are produced using other compounds, such as methanol, however the glycerol and oxygen uptake reactions are irreversible in the simulation model; or ii) that methanol is produced from glycerol and oxygen, but the utilization of methanol in fact consumes oxygen. In contrast, the EMs enable a rational interpretation of the flux data structure [6]. For instance, it can be deduced from the first principal EM, *e*_7_, that methanol is, under the consumption of oxygen and the release of carbon dioxide, mainly transformed to ethanol (a scenario that was simulated in the simulation case but is rather not observed in *Pichia* cultivations). The results obtained by the different method for the rotation of the PCA loadings *w*_2_, *w*_3_ and *w*_5_ are equal, suggesting their association to glycerol uptake, methanol uptake and CO_2_ release, respectively. Different results are given by the different methods for the rotations of the loadings *w*_1_ and *w*_4_. For *w*_1_ the results of the methods agree in so far as that glucose uptake is predicted in each case. The results of varimax, orthomax and quartimax in addition suggest that citrate release is also associated to this loading, wherefore this loading seems to be similar to the active *e*_13_. The results for loading *w*_4_ suggest a biomass growth association in case of varimax and orthomax, an oxygen uptake association by quartimax and parsimax and an association to ethanol secretion in case of equamax. Thus, despite the fact that the rotated loadings are easier to interpret than the original loadings, it is not clear in some cases to which reaction the loading is really associated, i.e. the methods yield different results. In comparison to the selected EMs, the rotated loadings do not, in general, seem to reproduce the correlation between substrate uptake and product secretion, hence they do not provide the same level of insight as the identified EMs.

#### Analysis of the impact of noise on the performance

The impact of noise on the EM selection was studied by adding 2 % or 10 % Gaussian noise to the simulated data. The respective performances of PEMA and PCA are shown in Table 2 of Additional file 1 and Table 2. In the case of 10 % Gaussian noise, the EMs identified with PEMA are identical to the case of no noise and also the explained variance values are very similar, differing by less than 1 %. Comparing the results obtained with PCA it can be seen that they are also very similar. The observation that the performance in terms of explained variance did not significantly deteriorate for both PEMA and PCA when adding noise to the data is partially due to the cancelation of noise when compressing the data via the loadings or EMs into a latent space.

**Table 2:** Selected EMs and the respective variance (ϑ) values for one to 10 number of *n*_*Fac*_s obtained for the simulated data with 10 % Gaussian noise

*n*_*Fac*_	EM/ϑ	1	2	3	4	5	6	7	8	9	10
1	EM	70									
1	ϑ	29.61									
2	EMs	70	7								
2	ϑ	29.61	54.09								
3	EMs	70	7	40							
3	ϑ	29.61	54.09	71.50							
4	EMs	7	69	13	40						
4	ϑ	24.60	47.20	65.78	83.55						
5	EMs	7	71	33	13	37					
5	ϑ	24.60	46.34	64.74	82.59	94.05					
6	EMs	7	33	13	3	37	23				
6	ϑ	24.60	44.17	62.35	79.05	91.47	97.09				
7	EMs	7	33	13	3	37	23	12			
7	ϑ	24.60	44.17	62.35	79.05	91.47	97.09	97.26			
8	EMs	7	33	13	3	37	23	12	19		
8	ϑ	24.60	44.17	62.35	79.05	91.47	97.09	97.26	97.30		
9	EMs	7	33	13	3	37	23	12	19	16	
9	ϑ	24.60	44.17	62.35	79.05	91.47	97.09	97.26	97.30	97.31	
10	EMs	7	33	13	3	37	23	12	19	16	17
10	ϑ	24.60	44.17	62.35	79.05	91.47	97.09	97.26	97.30	97.31	97.32
PCA	n_lv_**	1	2	3	4	5	6	7	8	9	
PCA	ϑ	50.04	82.08	91.83	97.05	99.39	99.96	100.00	100.00	100.00	

The set of truly active EMs for data generation was EMs = [1, 3, 7, 12, 13, 14, 16, 19, 20, 22, 23, 24, 28, 32, 33, 37]. *n*_*lv*_ ** number of latent variables for PCA

**Fig. 3:**
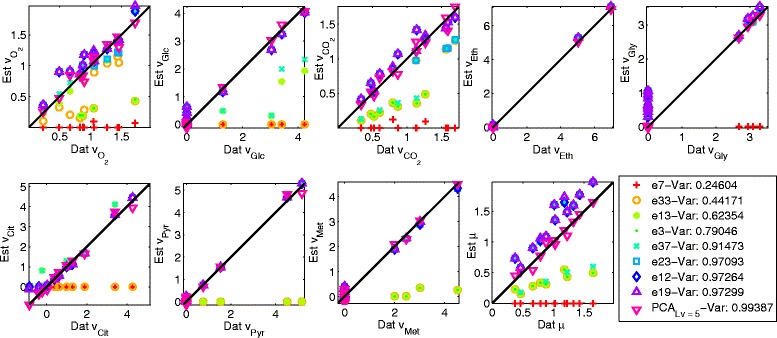
Normalized flux estimations over normalized flux data for PCA with five latent variables and each of the 11 identified EMs by PEMA in the case of 10 % Gaussian noise

The selection of the number of latent variables to be included into the PCA model can be nontrivial, because once all data underlying “true” patterns have been extracted, the PCA will start to model noise patterns. Typically, one analyzes the changes in captured variance to decide upon what number of latent variables to use. The choice of the number of factors in the case of PEMA seems to be easier. It can be seen that PEMA reaches a plateau at a value of 97.2 % of explained variance in both the cases no noise (Table 1) and 10 % noise (Table 2). Once PEMA extracted all main features in the data, the method will “choose” between the various EMs such that the patterns remaining in the data are explained. Though the method then starts to capture noise patterns as well, the changes in explained variance values are very low, less than those observed for PCA for the same amount of captured variance. The reason is that, unlike PCA, the entries of the EMs vectors are fixed a priori and not adapted to the data as in the case of the PCA loadings. However, in the case of 10 % noise, Table 2, the explained variance value increases by 0.01 % from nine to ten *n*_*Fac*_s, erroneously suggesting that the 10^th^ EM would also belong to the set of active EMs. Thus care must also be taken when choosing the number of PEMs. In the present study either a number of nine PEMs seems to be appropriate to describe the data and a number of four or five latent variables in the case of PCA. The estimations obtained with one to eight PEMs (PEMA) and five latent variables (PCA) are shown in Fig. 3 for the case of 10 % noise. The estimations of the fluxes improve for an increasing number of PEMs and also the differences in the contributions of the PEMs to the flux estimations can be noted. For all eight PEMs a good agreement between the data and estimations can be found. It suggests however that the zero values of the glucose, glycerol, citrate and methanol fluxes are not so well approximated. This might be due to the fact that these values do not have a big impact on the estimation performance. In the case of citrate it can also be observed that its uptake is not considered by the selected PEMs, as the negative flux data values are estimated to be zero. Again the reason for this might be the magnitude of these values and their low contribution to the estimation performance. In the case of PCA, the estimations match the data very well for all fluxes.

#### Analysis of the number of evaluated EMs combinations and computation time

**Fig. 4:**
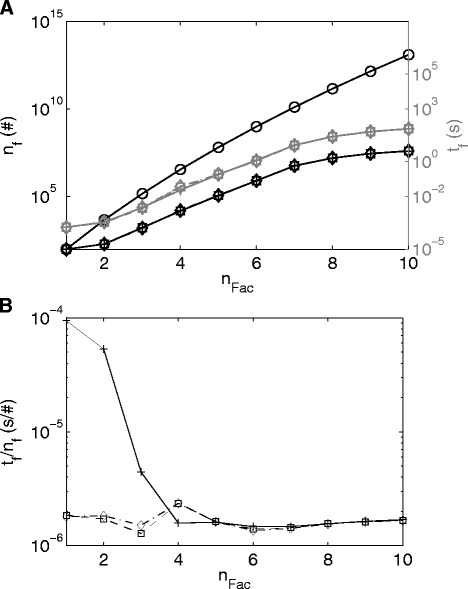
**a** Number of combinations of EMs, *n*_*f*_, on the left axis and computation time for the evaluation of the combinations, *t*_*f*_ on the right axis against numbers of factors, *n*_*Fac*_. *Black circle* and *continuous line*: Theoretical possible number of combinations; *Black/Gray plus* and *continuous line*: Combinations evaluated/Computation time by the branch and bound approach in case of no noise; *Black/Gray square* and *dashed line*: Combinations evaluated/Computation time by the branch and bound approach in case of 2 % noise; *Black/Gray star* and *dashed-dotted line*: Combinations evaluated/Computation time by the branch and bound approach in case of 10 % noise. **b** Computation time for the evaluation of one EM, *t*_*f*_/*n*_*f*_. *Black plus* and *continuous line*: No noise; *Black square* and *dashed line*: 2 % noise; *Black star* and *dashed-dotted line*: 10 % noise

The greater the number of *n*_*Fac*_s (and the greater the number of EMs) the greater the number of possible EM combinations that have theoretically to be evaluated, as shown in Fig. 4a. However, in the case of the proposed branch and bound method fewer combinations are evaluated for an increasing number of *n*_*Fac*_s, ranging from 5 % of evaluated to possible combinations for two factors to about 0.0005 % for 10 factors. The number of evaluated combinations varies slightly for different levels of noise, but the number of evaluations, for all levels, remains far below the theoretically feasible number of combinations, i.e.: at most 5 % of the theoretically possible combinations were evaluated. The computation time increases fairly linearly with the number of EMs combinations that were evaluated. On average it takes 1.57 × 10^−6^ s to evaluate how much variance is explained by one EM, Fig. 4b.

### Pichia pastoris experimental case study

This case uses the same metabolic network of *Pichia pastoris* as the simulated case, containing a set of 98 EMs [23]. The aim in this case study is to evaluate the performance of the PEMA under real experimental conditions, potentially revealing which metabolic pathways are active. The flux data used in this study stem from Tortajada et al’s collection of flux data from the literature [23]. The set of EMs also originated from Tortajada et al’s study [23], where they were analyzed with respect to possible substrate conversion to biomass and compared to experimental yield data. In Table 3 the EMs selected with the proposed approach and their respective explained variance values for different numbers of factors are shown. It can be seen that from four factors on, the same one to *n*_*Fac*_-1 EMs are chosen, which, in accordance with the findings in the simulated case study, indicating that a set of truly active EMs is identified. For the fourth factor two EMs are given, because *e*_4_ and *e*_22_ are indistinguishable given only the measured flux values (*e*_22_ was eliminated by the EM pre-selection). For more than six factors the explained variance value does not further significantly increase, which suggests that the EMs identified for greater number of factors might not be biologically significant, i.e. they are used to explain the noise patterns. The clear plateau in the variance values, which can be observed in all studied cases for PEMA, helps in the choice of the number of factors, as the results indicate that the appropriate number of EMs is the one, which explains the most variance before reaching the plateau. Thus, a set of six PEMs is identified, which can explain about 99 % of the variance observed in the data. The choice of the number of latent variables in the case of PCA seems to be harder than the choice of the number of PEMs in PEMA. While typically a number of latent variables that explains about 90 % of the variance would be chosen in the case of PCA (since the remaining variance is considered to be noise only), the variance values are still increasing significantly for an increasing number of latent variables (Table 3). Thus, two or three latent variables could be appropriate to explain the variation in the data with PCA.

**Table 3:** Selected EMs and the respective captured variance (ϑ) values for one to ten number of *n*_*Fac*_s obtained for experimental data given in Tortajada et al. [23] and the best first solution of the greedy approach (BF**)

*n*_*Fac*_	EM/ϑ	1	2	3	4	5	6	7	8	9	10
1	EM	90									
1	ϑ	42.32									
2	EMs	76	14								
2	ϑ	38.88	76.83								
3	EMs	76	14	40							
3	ϑ	38.88	76.83	92.45							
4	EMs	32	14	33	4/22						
4	ϑ	38.71	76.66	90.41	98.87						
5	EMs	32	14	33	4/22	37					
5	ϑ	38.71	76.66	90.41	98.87	98.95					
6	EMs	32	14	33	4/22	37	12				
6	ϑ	38.71	76.66	90.41	98.87	98.95	99.00				
7	EMs	32	14	33	4/22	37	12	1			
7	ϑ	38.71	76.66	90.41	98.87	98.95	99.00	99.01			
8	EMs	32	14	33	4/22	37	12	1	5		
8	ϑ	38.71	76.66	90.41	98.87	98.95	99.00	99.01	99.01		
9	EMs	32	14	33	4/22	37	12	1	5	19	
9	ϑ	38.71	76.66	90.41	98.87	98.95	99.00	99.01	99.01	99.01	
10	EMs	32	14	33	4/22	37	12	1	5	19	20
10	ϑ	38.71	76.66	90.41	98.87	98.95	99.00	99.01	99.01	99.01	99.01
PCA	n_lv_*	1	2	3	4/22	5	6	7	8	9	
PCA	ϑ	92.03	97.16	99.84	99.98	100.00	100.00	100.00	100.00	100.00	
BF**	EMs	89	75	39	4	12	20	1	5	10	7
BF**	ϑ	42.32	76.72	92.33	97.95	98.10	98.20	98.27	98.27	98.27	98.27

*n*_*lv*_ * number of latent variables for PCA

**Fig. 5:**
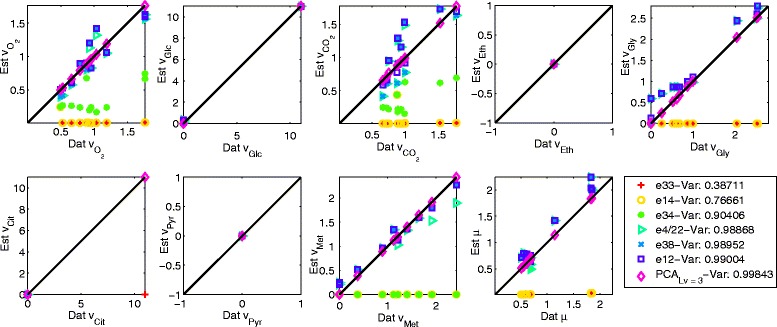
Normalized flux estimations over normalized flux data for PCA with three latent variables and each of the six identified EMs by PEMA

**Fig. 6:**
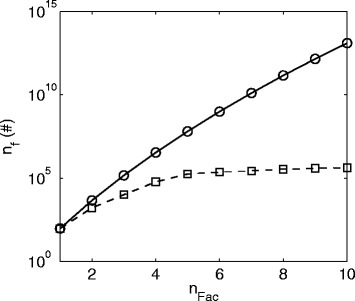
Number of combinations of EMs, *n*_*f*_, over numbers of factors, *n*_*Fac*_. *Black circle* and *continuous line*: Theoretical possible number of combinations; *Black squares* and *continuous line*: Combinations evaluated by the branch and bound approach

The estimations obtained with one to six EMs (PEMA) and the three latent variables (PCA) for the flux data are shown in Fig. 5. The contributions of the selected PEMs to each of the flux estimations can be clearly observed and the flux estimations with six PEMs are close to the experimental flux data. In case of the carbon dioxide and oxygen fluxes some of the estimations do not approximate the data well, which is most probably due to the overall magnitude of the carbon dioxide and oxygen flux values (which are rather low compared to the other fluxes) and consequently their low impact on performance. Also slight mismatches can be observed in the case of low glycerol flux values, most probably due to the same reason. The PCA estimations match the data very well for all fluxes. The number of evaluated EM combinations is, as in the simulated case, much lower than the theoretically possible number of combinations ranging from 36 % (evaluated/theoretically possible) with two factors to 3.36 × 10^−6^ % with ten factors, see Fig. 6.

#### Biological interpretation of the PEMs

**Fig. 7:**
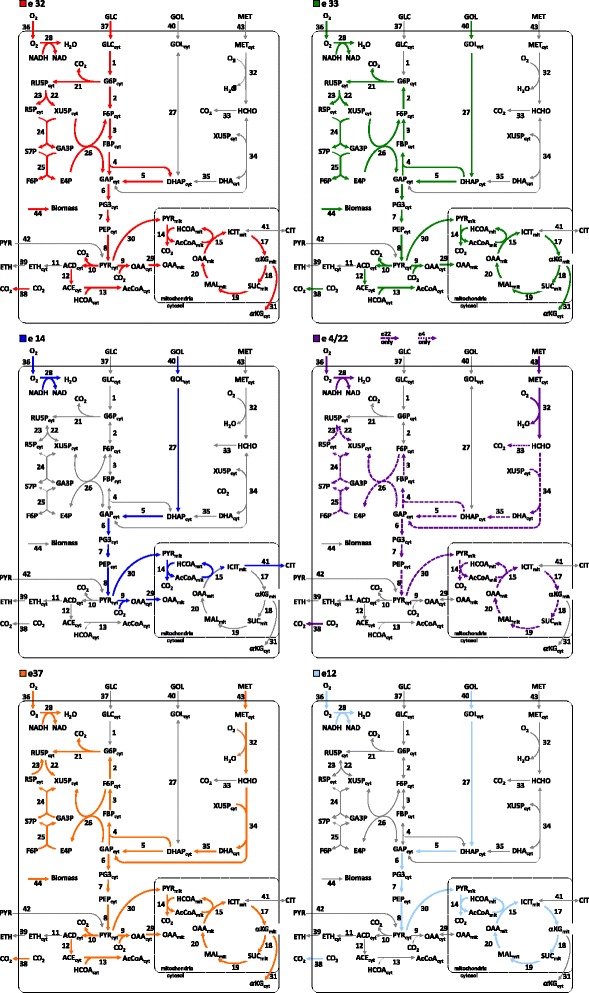
The metabolic network of *Pichia pastoris* considered in this study and adopted from Tortajada et al. [23]. The different *colored arrows* correspond to the PEMs of the experimental case study

The advantage of PEMA is that the predominantly active pathways can be identified by looking at the selected PEMs. The metabolic network, which was adopted from Tortajada et al. [23] and used in this study, is shown in Fig. 7, together with the predominantly active pathways as indicated by the PEMs which are represented in different colors. It can be seen that *e*_32_, *e*_33_ and *e*_37_ describe biomass growth using either glucose, glycerol or methanol, respectively. These three EMs have the shortest paths for growth of biomass on the respective substrate while also adhering to the secretion rate constraints, i.e. the shortest distance between biomass and the respective substrate, where the length of a path/EM is the number of reactions that it comprises. Shorter distances between two compounds are favored from an evolutionary point of view [26, 27] and the selected EMs such seem to make sense. Also shorter EMs can carry higher fluxes [9], which in case of the three selected EMs might allow higher growth rates.

**Fig. 8:**
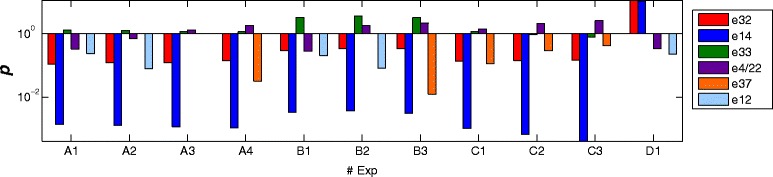
Intensities of the scaled weights, *p*, for each identified EM and for each of the experiments are shown. In all experiments vGlc = 0 (reaction 37) and vCit = 0 (reaction 41) except for D1. vMet = 0 (reaction 43) in experiments A1 and B1. vGly = 0 (reaction 40) in C3 and D1. vEth = 0 for all experiments. For more details see Tortajada et al. [23]

The *e*_14_ describes the uptake of glycerol and release of citrate and is involved in the reduction of NAD+ to NADH. It is the shortest EM for production of citrate from glucose, which again might mean that this path is evolutionary favored [26, 27]. As can be taken from Fig. 8, this EM is inactive for most experiments. In the ambiguous case of *e*_4_/*e*_22_ (Fig. 7) methanol is consumed using oxygen thereby releasing carbon dioxide. Both EMs seem to be involved in the generation of reducing equivalents, reducing NAD+ to NADH via reactions 33 or 14, either in the cytosol (*e*_4_) or mitochondria (*e*_22_), respectively. However, the length of EM4 of 6 is significant shorter than *e*_22_ with 25, wherefore it could be hypothesized that *e*_4_ is more likely to be “really” active. The production of energy and NADH from glycerol is described by *e*_12_, which has a length of 16. For reactions 2, 3 and 4, the direction of *e*_32_ is opposed to *e*_33_ and *e*_37_, which seems to violate the non-cancelation principle [5, 23]. However, looking at the weights in Fig. 8, it can be observed that the opposing contributions of the EMs generally differ by one order of magnitude. For different experiments the EMs are weighted differently, i.e. a distinct EM activity pattern can be observed for every experiment. Thus, it seems that for each experiment the cancelation principle is retained. Other opposing contributions of EMs to reactions in the pentose phosphate pathway, which were analyzed in the same way, also seem experimentally to adhere to the cancelation principle. In future, the introduction of a hard constraint into the branch and bound part of the algorithm that accounts explicitly for the cancelation principle might help to reduce the number of evaluations of EM combinations further.

None of the PEMs is predominantly active for all experiments (Fig. 8). This might be due to the algorithm, which requires sufficient excitation/variation in the data for the identification of the EMs, whereas constantly active EMs might show a rather low variation. However, the variations in the activity patterns might be due to changes in the cellular environment. For instance, experiment D1 was the only one in which glucose uptake and citrate secretion were present, which is reflected by much greater activity of *e*_14_ than in the other experiments. Therefore, it seems that the cell responds to the changing environment by regulation of the activity of different pathways, reflected by the activity of the different EMs, as is captured by PEMA.

### Saccharomyces cerevisiae experimental case study

A metabolic network for *Saccharomyces cerevisiae* proposed by Hayakawa et al. [28] and fluxome data from [28] and [29] was used in this study (Additional file 4 contains both data sets). The network describes the central cytosolic and mitochondrial metabolism of *S. cerevisiae*, comprising glycolysis, the pentose phosphate pathway, anaplerotic carboxylation, fermentative pathways, the TCA cycle, malic enzyme and anabolic reactions from intermediary metabolites into anabolism [28, 29]. A biomass synthesis reaction was incorporated from [30] replacing the single reactions for every biomass component, in order to bundle the flux contributions for biomass growth (see Additional file 4). The network contains 42 compounds (30 of which are internal metabolites, which can be balanced for growth) and 47 reactions, yielding a total number of 1182 EMs, which were calculated using the EFM toolbox [31].

**Fig. 9:**
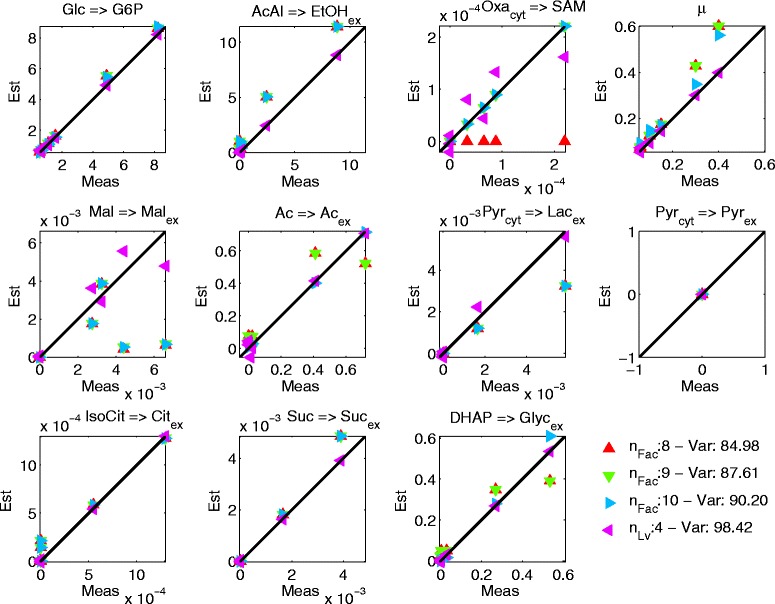
Reaction estimations over data of the exchange reactions for PCA with five latent variables and for PEMA with eight, nine and ten factors. The units of the reactions are in mmol/gCDW/h except for specific biomass growth 1/h. The corresponding reactions are shown on the *top*, where => signifies irreversible reactions

The objective in this case study is to evaluate the performance of PEMA on fluxome data and for a case with a greater number of EMs, i.e. 1182 EMs in this case in comparison to 98 EMs in the prior cases. The observed behavior in the explained variance for one to six factors is similar to the one observed in the other case studies, i.e. a shift from an initial selection of the same 1 to *n*_*Fac*_-1 EMs (1–3 factors) to a second selection of the same EMs for four to six factors (Table 4). However, in the present case the combination of selected EMs changes several times for further increases in the number of factors. Only for eight and nine factors the same 1 to *n*_*Fac*_-1 EMs are selected again. For nine factors all reactions are represented for the first time in the selected EMs, as can e.g. be seen in Fig. 9 (all results can be found in the Additional file 1), wherefore nine factors seem to provide a minimal base. However, the explained variance value increases by 2.6 % from nine to ten factors, which in comparison to the behavior observed in the cases before, seems to indicate that more than nine factors should be chosen. The percentage of the explained variance of the greedy solution is found to stabilize around 91 % from ten factors on (Table 4) and the explained variance seems to converge towards 92 % for an increasing number of factors, which agrees with the behavior observed in the previous cases. The computation time required for the evaluation of combinations with 11 factors stalled as described later, which is most likely due to the very low differences in the explained variance values between different combinations of EMs. Hence, it might be that the increase in the explained variance value from ten factors on is describing noise rather than the underlying behavior. For PCA, the total explained variance is slightly higher than in case of PEMA, which matches the observation in the other cases. Three or four latent variables can be chosen for PCA.

**Table 4:** Selected EMs and the respective captured variance (ϑ) values for one to ten number of *n*_*Fac*_s obtained for experimental data extracted from [28] and [29]

*n*_*Fac*_	EM/ϑ	1	2	3	4	5	6	7	8	9	10	11	12	13
1	EMs	915												
1	ϑ	28.12												
2	EMs	915	1145											
2	ϑ	28.12	46.44											
3	EMs	678	1145	988										
3	ϑ	25.54	44.93	56.05										
4	EMs	1145	663	968	750									
4	ϑ	22.62	41.62	55.31	65.07									
5	EMs	1145	663	968	750	48								
5	ϑ	22.62	41.62	55.31	65.07	72.62								
6	EMs	1145	663	968	750	48	31							
6	ϑ	22.62	41.62	55.31	65.07	72.62	77.23							
7	EMs	1145	663	766	968	48	31	1047						
7	ϑ	22.62	41.62	55.33	65.05	72.61	77.22	81.15						
8	EMs	1145	663	750	48	647	718	31	1040					
8	ϑ	22.62	41.62	55.25	62.80	69.99	76.42	81.03	84.98					
9	EMs	1145	663	750	48	647	718	31	1040	641				
9	ϑ	22.62	41.62	55.25	62.80	69.99	76.42	81.03	84.98	87.61				
10	EMs	1145	972	697	750	48	685	31	999	659	501			
10	ϑ	22.62	40.25	51.57	62.43	69.99	75.60	80.21	83.94	87.57	90.20			
PCA	#Lv	1	2	3	4	5	6							
PCA	n_lv_**	68.44	84.28	94.82	98.42	99.53	100.00							
BF*	EMs	893	1123	675	1115	26	13	1022	637	630	672	33	1155	1117
BF*	ϑ	28.12	46.44	55.71	64.85	72.40	77.01	80.69	84.14	86.77	89.19	90.42	91.29	91.73

*n*_*lv*_ ** number of latent variables for PCA. BF* best first solution obtained by the greedy approach

The estimated flux values for the exchange reactions with eight, nine and ten factors in case of PEMA and for PCA with four latent variables are shown in Fig. 9 (for the other reactions the plots can be found in the Additional file 1). The fit of the estimates to the experimental data is generally good for all these numbers of factors, however SAM production is only modeled from nine factors on. The greatest discrepancies can be observed in case of the malate reaction for both PEMA and PCA. With ten factors PEMA estimates the acetate and glycerol reactions significantly better than with nine factors, thus the PEMA solution with ten factors is preferred over the one with nine factors.

#### Biological interpretation of the PEMs

**Fig. 10:**
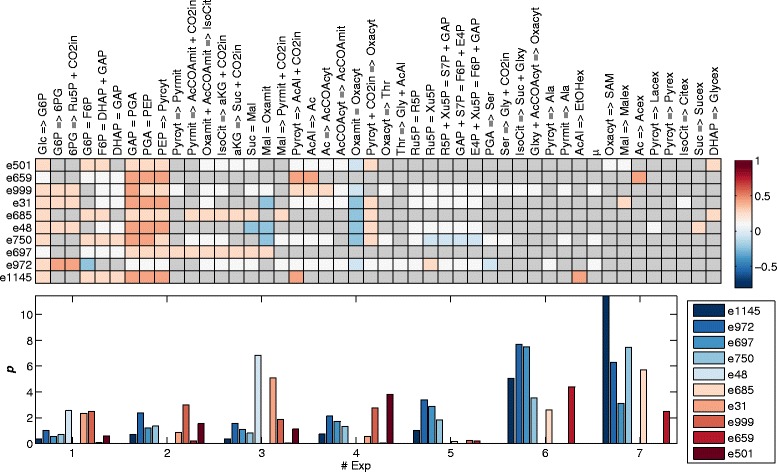
The entries in the active EMs selected by PEMA with ten factors for the reactions. Zero valued entries are shown in *grey*. The contributions of the active EMs to the seven experiments. Experiments 1 to 4: Strains S288C and Kyokai 6 at specific growth rates 0.06 and 0.1 (1/h) [28] and Experiments 5 to 7: Strain ATCC 32167 at specific growth rates 0.15, 0.3 and 0.4 (1/h) [29]

The active EMs and their contributions to each experiment are shown in Fig. 10. The conversion of glucose to ethanol described by *e*_1145_ was repeatedly selected by PEMA across different factors. This EM has a particularly high contribution in experiments six and seven, which were performed at high glucose consumption rates. It has a length of nine, which is the shortest EM for the conversion of glucose to ethanol. The shortest EM is also selected for transforming glucose into acetate, *e*_659_. This EM is active in experiments one, two and five to seven. The contributions are particularly high in experiments six and seven. As mentioned before, shorter EMs are assumed to be evolutionary favored [26, 27] and they can carry greater fluxes [9].

Three EMs, *e*_972_, *e*_750_ and *e*_999_, describe the conversion of glucose to biomass growth via different routes. The lengths of the EMs are 28, 27 and 30, respectively. The shortest EMs for biomass growth have a length of 26, the longest are 31, and the average length is 29. In experiments five to seven *e*_999_ is not active. Since the yield of biomass as a function of glucose varies between the experiments, two/three EMs are required to describe biomass growth. This also explains the variation in the length of the three selected EMs, which results in different yields of biomass growth on glucose of EMs, *e*_972_, *e*_750_ and *e*_999_, i.e.: 0.19, 0.37 and 0.27, respectively.

Energy generation is described by *e*_697_, which seems to be more active in experiments five to seven (experiments with strain ATCC 32167). The conversion of glucose to lactate and succinate, *e*_48_, can be observed to be predominantly active in experiments one and three, which both have a low specific biomass growth rate. Lactate and succinate formation were assumed to be zero for experiments five to seven and at the same time *e*_48_ is hardly active. This EM is the only one which transforms glucose to lactate and succinate and it is interesting to note that the flux goes through the pentose phosphate pathway. It was described in [29] that during oxidative growth the pentose phosphate pathway alone is sufficient to completely supply NADPH for anabolism, which might explain why the flux goes through this pathway.

The conversion of glucose to glycerol and the generation of energy are described by *e*_685_, which due to the involvement in both processes has a length of 15 and such is longer than EMs that only describe glucose conversion. This EM is majorly active in experiments six and seven, in agreement with the observed higher glycerol formation. The *e*_31_ was repeatedly selected by PEMA across different numbers of factors and it describes the formation of citrate and malate from glucose. The flux goes through the pentose phosphate pathway, thus it is longer (length 23) than the shortest option (length 21). In experiments five to seven citrate and malate formation was assumed to be zero and consequently this EM is not active for those experiments.

The production of S-adenosyl-L-methionine (SAM), malate and glycerol is described by *e*_501_. This EM is particularly active in experiments three and four, the experiments with the high SAM producing strain Kyokai 6. The increased activity of the TCA cycle observed in these experiments [28], is partially reflected by *e*_501_, in that malate is produced via the TCA cycle.

#### Analysis of the number of evaluated EMs combinations and computation time

**Fig. 11:**
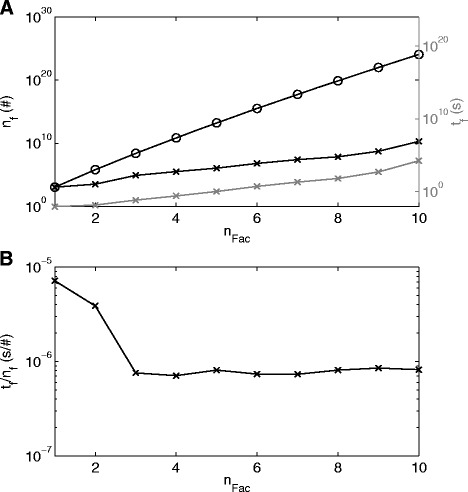
**a** Number of combinations of EMs, *n*_*f*_., on the *left* axis and computation time for the evaluation of the combinations, *t*_*f*_, on the *right* axis over numbers of factors, *n*_*Fac*_. *Black circle* and *continuous line*: Theoretical possible number of combinations; *Black/Gray plus* and *continuous line*: Combinations evaluated/Computation time by the branch and bound approach. **b** Computation time for the evaluation of one EM, *t*_*f*_/*n*_*f*_

The number of theoretically possible combinations of EM increased significantly due to the greater number of 1182 EMs opposed to 98 EMs in the cases before, i.e. 1.17 × 10^24^ (Fig. 11a) opposed to 1.26 × 10^+13^ (Fig. 4a) for 10 factors, respectively. The number of evaluated combinations and the computation of the branch and bound method also increased in the present case, but the average time to evaluate how much variance is explained by one combination decreased to 7.94 × 10^−7^ s in comparison to 1.57 × 10^−6^ s in the simulated case, Fig. 4b and Fig. 11b (Note that the average time is only computed for *n*_*Fac*_ > 4, since before this the time values are too low and they do not reflect the evaluation of the EMs but rather other effects, e.g. memory allocation). Thus, in this case the combinations are evaluated about 2 times faster (1.57 × 10^−6^/7.94 × 10^−7^). The reason for this improvement in performance is the way the algorithm can be implemented, using vector and matrix multiplications. Only two operations are required to evaluate *m* − *i*_*Fac*_ EMs, i.e.: computing the results for eqs. (5) and (8). Thus, it can be expected that in cases of a greater number of EMs a solution can still be obtained with reasonable computation time. However, critical for the computation time is increases in the number of factors, as can be seen in Fig. 11a. This also becomes evident looking at the theoretically possible number of EM combinations:

10 $$ {n}_{comb}=\frac{m!}{n_{Fac}!\cdot \left(m-{n}_{Fac}\right)!}=\frac{{\displaystyle {\prod}_{i=1}^{n_{Fac}}}\left(m-\left(i-1\right)\right)}{n_{Fac}!}\approx \frac{m^{n_{Fac}}}{n_{Fac}!} $$

The increase in the number of factors results in an exponential increase in the number of combinations. The numbers of evaluated combinations do increase much more slowly, however the number of evaluated combinations and computation time for more than ten factors are not shown, since the computation time exceeded two weeks. The reason for the drastic increase in computation time from ten to 11 factors is most likely that the differences in the explained variance values between the combinations is not as distinct as in the previous cases, wherefore many more combinations need to be evaluated and compared. This assumption is also supported by the observation that the explained variance values produced by the greedy approach stabilize around 91 % from ten factors on (Table 4). Thus, the proposed branch and bound approach seems to work efficiently even for greater number of EMs as long as the differences in the explained variance values for a given number of factors are sufficiently distinct. However, Increases in the number of EMs by several orders of magnitude have not been studied here and it might be that even with the proposed branch and bound approach the number of evaluations is so elevated that the application of approximation techniques, such as relaxation, becomes necessary.

## Conclusions

A method that analyzes reaction flux data using combinations of elementary (flux) modes (EM) has been proposed. The method avoids the evaluation of all possible combinations of EMs by using a branch and bound approach. It was shown that PEMA identifies the principal elementary modes (PEMs), which are those combinations of EMs that account for most of the variance in the flux data, and that PEMs are a faithful representation of active pathways. From studies in which 2 and 10 % Gaussian noise was added to the data, it can be concluded that the performance did not deteriorate for the correct identification of the PEMs. Also the performance in terms of explained variance did not decrease significantly for increasing levels of noise. In comparison to PCA it was observed that PCA can explain more variance in the data with fewer latent variables, but in contrast to PCA latent structures, the PEMs have a biological meaning. It also appears to be easier to choose the number of PEMs than the number of principal components in PCA. In addition, it was shown that the analysis of the PEMs might reveal insights into the regulation of the pathways. The set of PEMs is not exhaustive as only those PEMs can be identified that have a traceable footprint in the flux data, whereas other EMs might be active that do not contribute to the footprint significantly and thus are probably of minor interest.

## Declaration

### Ethics approval and consent to participate

Not applicable.

### Consent for publication

Not applicable.

### Availability of data and material

All data are publically available, either from the additional files or the original articles, which are cited in this article.

## Additional files

Additional file 1: Additional mathematical details of the algorithm, details for the generation of the simulation case data and additional results for the simulated *Pichia pastoris* and *Saccharomyces cerevisiae* case study (PDF 5250 kb) Additional file 2: Matlab implementation of the proposed algorithm. (RAR 125 kb) Additional file 3: Data of the simulated *Pichia pastoris* case study. (XLSX 57 kb) Additional file 4: Data of the *Saccharomyces cerevisiae* case study. (XLSX 15 kb)

## Abbreviations

EM: elementary flux mode
PCA: principal component analysis
PEM: principal elementary flux mode
PEMA: principal elementary mode analysis
SAM: S-adenosyl-L-methionine
