# Supplementary material for: A principal components method constrained by elementary flux modes: analysis of flux data sets
Source: BMC Bioinformatics. 2016 May 4;17:200. doi: 10.1186/s12859-016-1063-0 (PMC4855838; doi:10.1186/s12859-016-1063-0)
Supplement: Additional file 1: — Additional mathematical details of the algorithm, details for the generation of the simulation case data and additional results for the simulated Pichia pastoris and Saccharomyces cerevisiae case study (PDF 5250 kb) [file 12859_2016_1063_MOESM1_ESM.pdf]

Supplementary Material: A principal components method constrained by elementary flux modes: Analysis of flux data sets

Moritz von Stosch (1), Cristiana Rodrigues de Azevedo (1), Mauro Luis (1), Sebastiao Feyo de Azevedo (2), Rui Oliveira* (1)

1 – REQUIMTE/DQ, Faculty of Science and Technology, University Nova de Lisboa, Campus de Caparica, 2829-516 Caparica, Portugal
2 – DEQ, Faculty of Engineering, University do Porto, Rua Dr. Roberto Frias s/n, 4200-465 Porto, Portugal
*Corresponding author

A: The iterative calculation of the p's and its relation to the Gram Schmidt orthonormalization

For two normalized EMs, $e_{j,n}$ and $e_{k,n}$, the procedure described in the article for calculating the respective weightings from the flux matrix V_{mes} yields:

$$p_{j,n} = {e_{j,n}}^T \cdot V_{mes} \;, \tag{A 1}$$

such that:

$$V_{j,n} = e_{j,n} \cdot p_{j,n} \tag{A 2}$$

and

$$V_{\Delta mes} = V_{mes} - V_{j,n} \tag{A 3}$$

wherefore:

$$p_{k,n} = {e_{k,n}}^T \cdot V_{\Delta mes} \;. \tag{A 4}$$

Inserting the introduced relations in this equation yields:

$$p_{k,n} = {e_{k,n}}^T \cdot \left(V_{mes} - V_{j,n}\right) = {e_{k,n}}^T \cdot \left(V_{mes} - e_{j,n} \cdot p_{j,n}\right) \tag{A 5}$$

and further:

$$p_{k,n} = {e_{k,n}}^T \cdot \left(V_{mes} - e_{j,n} \cdot \left({e_{j,n}}^T \cdot V_{mes}\right)\right) . \tag{A 6}$$

Reformulating:

$$p_{k,n} = \left({e_{k,n}}^T - {e_{k,n}}^T \cdot e_{j,n} \cdot {e_{j,n}}^T\right) \cdot V_{mes} \tag{A 7}$$

Introducing the relation

$$p_{k,n} = {e_{k,c,n}}^T \cdot V_{mes} \tag{A 8}$$

Where $e_{k,c,n}$ could be interpreted as the interference corrected $e_{k,n}$, yields:

$${e_{k,c,n}}^T \cdot V_{mes} = \left({e_{k,n}}^T - {e_{k,n}}^T \cdot e_{j,n} \cdot {e_{j,n}}^T\right) \cdot V_{mes} \tag{A 9}$$

and rearrangement

$${e_{k,c,n}}^T = {e_{k,n}}^T - {e_{k,n}}^T \cdot e_{j,n} \cdot {e_{j,n}}^T \quad , \tag{A 10}$$

which is equal to what the Gram Schmidt orthonormalization for the normalized EMs would yield in case that the weight values of $p_{j,n}$ and $p_{k,n}$ are greater than zero. The same can be shown for more factors. The orthonormalization of the elementary modes in the proposed method is implicitly achieved by the subtraction of the captured variance from the data, Eq. (A3), and subsequent analysis of the variance remaining in the data, Eq. (A4,) as in principle component analysis. This is an important feature, as this means that:

i) the calculated contributions can simply be summed since they are independent;

ii) the iteratively calculated variances for the EMs can be summed (see also Eq. (B6)), wherefore:

$$\left(\sum_l p_{l,n}\right)^2 = \sum_l \left(p_{l,n}\right)^2, \qquad \text{(A 11)}$$

which allows us to simplify equations (7) resulting in equation (8), see paper;

iii) allows us to assess how much information two EMs share, see following.

B: Analysis of the interference between two successive EMs

The flux patterns which are captured by the combination of the two normalized EMs, $e_{j,n}$ and $e_{k,n}$, is described by the sum:

$$V_{est} = V_{j,n} + V_{k,n} \qquad \text{(B 1)}$$

When inserting the relations presented before

$$V_{est} = V_{j,n} + V_{k,n} = e_{j,n} \cdot p_{j,n} + e_{k,n} \cdot p_{k,n} \quad , \qquad \text{(B 2)}$$

$$V_{est} = e_{j,n} \cdot \left({e_{j,n}}^T \cdot V_{mes}\right) + e_{k,n} \cdot \left({e_{k,n}}^T \cdot V_{\triangle mes}\right) \quad , \qquad \text{(B 3)}$$

$$V_{est} = e_{j,n} \cdot \left(e_{j,n}{}^T \cdot V_{mes}\right) + e_{k,n} \cdot \left(e_{k,n}{}^T \cdot (V_{mes} - V_{j,n})\right) \quad , \tag{B 4}$$

$$V_{est} = e_{j,n} \cdot \left(e_{j,n}{}^T \cdot V_{mes}\right) + e_{k,n} \cdot \left(e_{k,n}{}^T \cdot \left(V_{mes} - e_{j,n} \cdot \left(e_{j,n}{}^T \cdot V_{mes}\right)\right)\right) , \tag{B 5}$$

and rearranging

$$V_{est} = e_{j,n} \cdot \left(e_{j,n}{}^T \cdot V_{mes}\right) + e_{k,n} \cdot \left(e_{k,n}{}^T \cdot V_{mes}\right) - \left(e_{k,n}{}^T \cdot e_{j,n} \cdot \left(e_{j,n}{}^T \cdot V_{mes}\right)\right) . \tag{B 6}$$

If the inner vector product of $e_{k,n}{}^T \cdot e_{j,n}$ would be zero then the contributions would be independent, i.e.:

$$e_{k,n}{}^T \cdot e_{j,n} = \left|e_{k,n}\right| \cdot \left|e_{j,n}\right| \cdot \cos\left(\sphericalangle e_{k,n}, e_{j,n}\right) = 0 \qquad .$$

Wherefore the last term in equation (B 6) would vanish. Otherwise the subtracted term is the information, which both EMs share and, if it would not be subtracted, would contribute two times to the summation of flux pattern. Note that it is exactly the contribution of this term, which is eliminated by the Gram-Schmidt approach through orthonormalization and in the proposed algorithm in implicitly accounted for by the subtraction of the captured variance from the data, Eq. (A3), and subsequent analysis of the variance remaining in the data, Eq. (A4). The decompositions of V_{mes} using e_j and then e_k are thus independent!

C: Pichia pastoris Simulation Case

This simulation case study is based on the metabolic network of *Pichia pastoris* proposed by Tortajada et al [1] and the provided 98 EMs are herein used for flux data generation. Twelve different experimental conditions were simulated, see Table 1. Considering all of the different conditions the EMs were filtered employing the following rational. If a compound is not present and cannot be produced then its contribution in the EM must be zero. If a compound is present

and is consumed then it cannot be secreted at the same time. The reduced set of EMs possibly active considering these scenarios comprised 76 EMs. The rank of the reduced set of EMs was analyzed (rank=20) and from the reduced set a subset of EMs was chosen that spans the space of (provides a basis to) the reduced set (20 EMs). For each scenario only those EMs (out of the subset of the reduced set) that can be active were assumed to contribute randomly to the flux pattern, but obeying to the minimum and maximum values of the fluxes given in Tortajada et al[1]. In total only 16 EM were active, namely EMs=[1, 3, 7, 12, 13, 14, 16, 19, 20, 22, 23, 24, 28, 32, 33, 37].

Table 1: Biomass growth in different simulated experimental scenarios

Exp	Experimental Scenario	Condition for fluxes
1	Growth on Glucose	vGly=0, vPyr=0, vMet=0
2	Growth on Methanol	vGlc=0, vGly=0, vPyr=0
3	Growth on Glycerol	vGlc=0, vPyr=0, vMet=0
4	Growth on Glucose and Pyruvate	vGly=0, vMet=0
5	Growth on Methanol and Pyruvate	vGlc=0, vGly=0
6	Growth on Glycerol and Pyruvate	vGlc=0, vMet=0
7	Growth on Glucose without Ethanol formation	vEth=0, vGly=0, vPyr=0, vMet=0
8	Growth on Methanol without Ethanol formation	vEth=0, vGlc=0, vGly=0, vPyr=0
9	Growth on Glycerol without Ethanol formation	vEth=0, vGlc=0, vPyr=0, vMet=0
10	Growth on Glucose and Pyruvate without Ethanol formation	vEth=0, vGly=0, vMet=0
11	Growth on Methanol and Pyruvate without Ethanol formation	vEth=0, vGlc=0, vGly=0
12	Growth on Glycerol and Pyruvate without Ethanol formation	vEth=0, vGlc=0, vMet=0

D: Results Simulation Case – Impact of Noise on the EM identification

Table 2: Selected EMs and the respective captured variance (ϑ) values for one to 10 number of n_{Fac}s obtained for the simulated data with 2% Gaussian noise. The set of truly active EMs for data generation was EMs=[1, 3, 7, 12, 13, 14, 16, 19, 20, 22, 23, 24, 28, 32, 33, 37]. n_{lv}**: Number of latent variables for PCA.

n_{Fac}	EM/ϑ	1	2	3	4	5	6	7	8	9	10
1	EM	70									
1	ϑ	29.39									
2	EMs	70	7								

2	ϑ	29.39	53.80								
3	EMs	70	7	40							
3	ϑ	29.39	53.80	71.13							
4	EMs	7	69	13	40						
4	ϑ	24.53	46.87	65.83	83.53						
5	EMs	7	71	13	33	37					
5	ϑ	24.53	45.98	64.40	82.47	93.95					
6	EMs	7	33	13	3	37	23				
6	ϑ	24.53	44.08	62.34	78.92	91.37	97.07				
7	EMs	7	33	13	3	37	23	12			
7	ϑ	24.53	44.08	62.34	78.92	91.37	97.07	97.21			
8	EMs	7	33	13	3	37	23	12	19		
8	ϑ	24.53	44.08	62.34	78.92	91.37	97.07	97.21	97.25		
9	EMs	7	33	13	3	37	23	12	19	16	
9	ϑ	24.53	44.08	62.34	78.92	91.37	97.07	97.21	97.25	97.26	
10	EMs	7	33	13	3	37	23	12	19	16	17
10	ϑ	24.53	44.08	62.34	78.92	91.37	97.07	97.21	97.25	97.26	97.26
PCA	n_{lv}**	1	2	3	4	5	6	7	8	9	
PCA	ϑ	50.13	82.14	91.80	96.98	99.27	99.96	100.00	100.00	100.00	

E: Saccharomyces cerevisiae experimental case study – Reaction Network

The metabolic network proposed by Hayakawa et al [2] was adopted in this study. To improve the performance of the algorithm the biomass synthesis equation of Gianchandani et al [3] was used instead of the variable biomass synthesis rates used in [2]. Consistency of the metabolic model and data was verified using Metabolic Flux Analysis. The system was observed to be redundant and consistent. The data and the network reactions can be found in an excel-file in the Supplementary Material.

F: Saccharomyces cerevisiae experimental case study – Additional Results

Estimations of reactions for PEMA with different numbers of factors and PCA

The plots of the estimated reaction over the measured values for different numbers of factors is in the following shown for all reactions. The legend in the last plot contains the information of the correspondences between color/symbols and the number of factors.

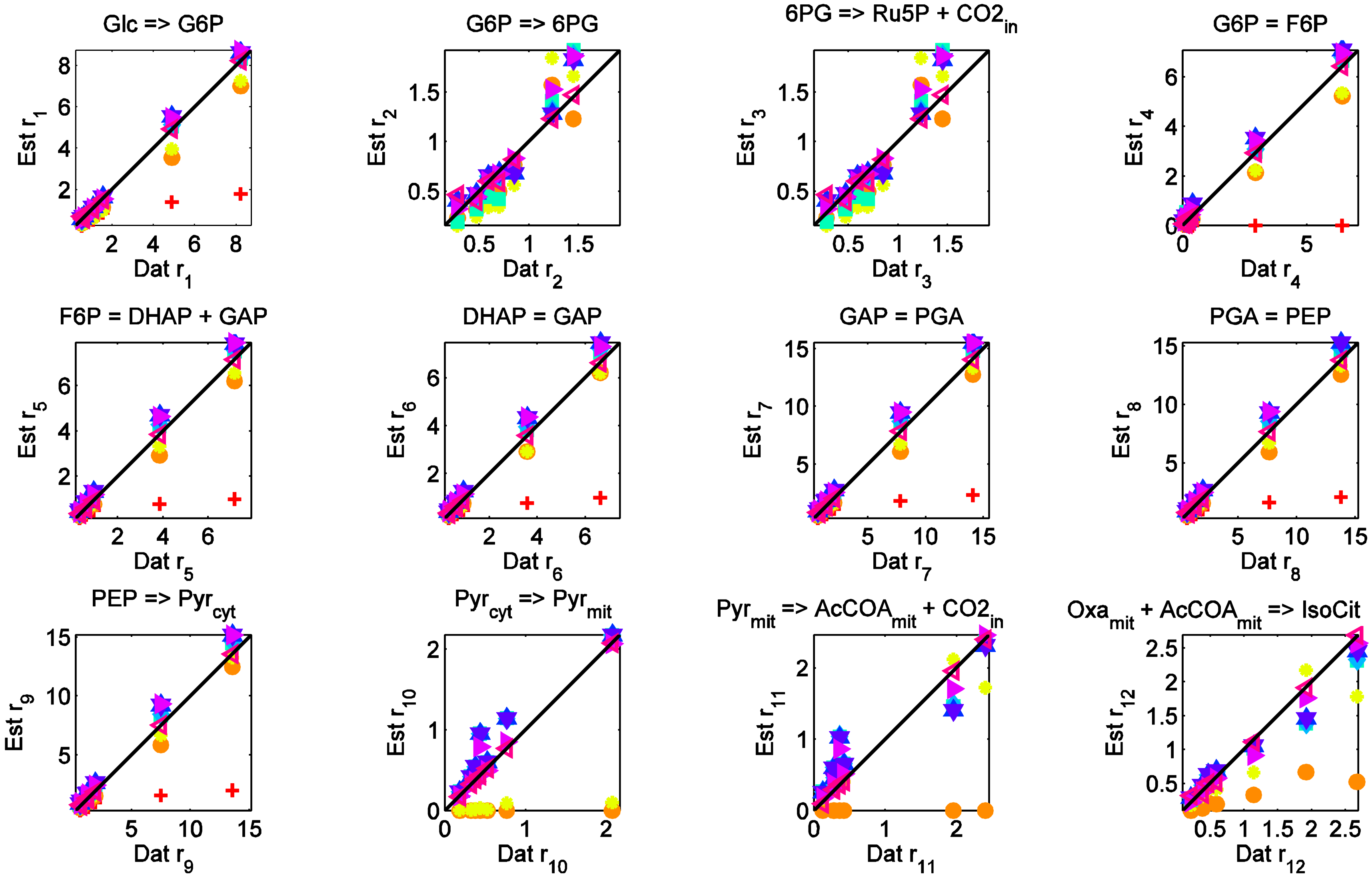

Glc => G6P
G6P => 6PG
6PG => Ru5P + CO2in
G6P = F6P
F6P = DHAP + GAP
DHAP = GAP
GAP = PGA
PGA = PEP
PEP => Pyrcyt
Pyrcyt => Pyrmit
Pyrmit => AcCOAmit + CO2in
Oxamit + AcCOAmit => IsoCit
Est r1
Dat r1
Est r2
Dat r2
Est r3
Dat r3
Est r4
Dat r4
Est r5
Dat r5
Est r6
Dat r6
Est r7
Dat r7
Est r8
Dat r8
Est r9
Dat r9
Est r10
Dat r10
Est r11
Dat r11
Est r12
Dat r12

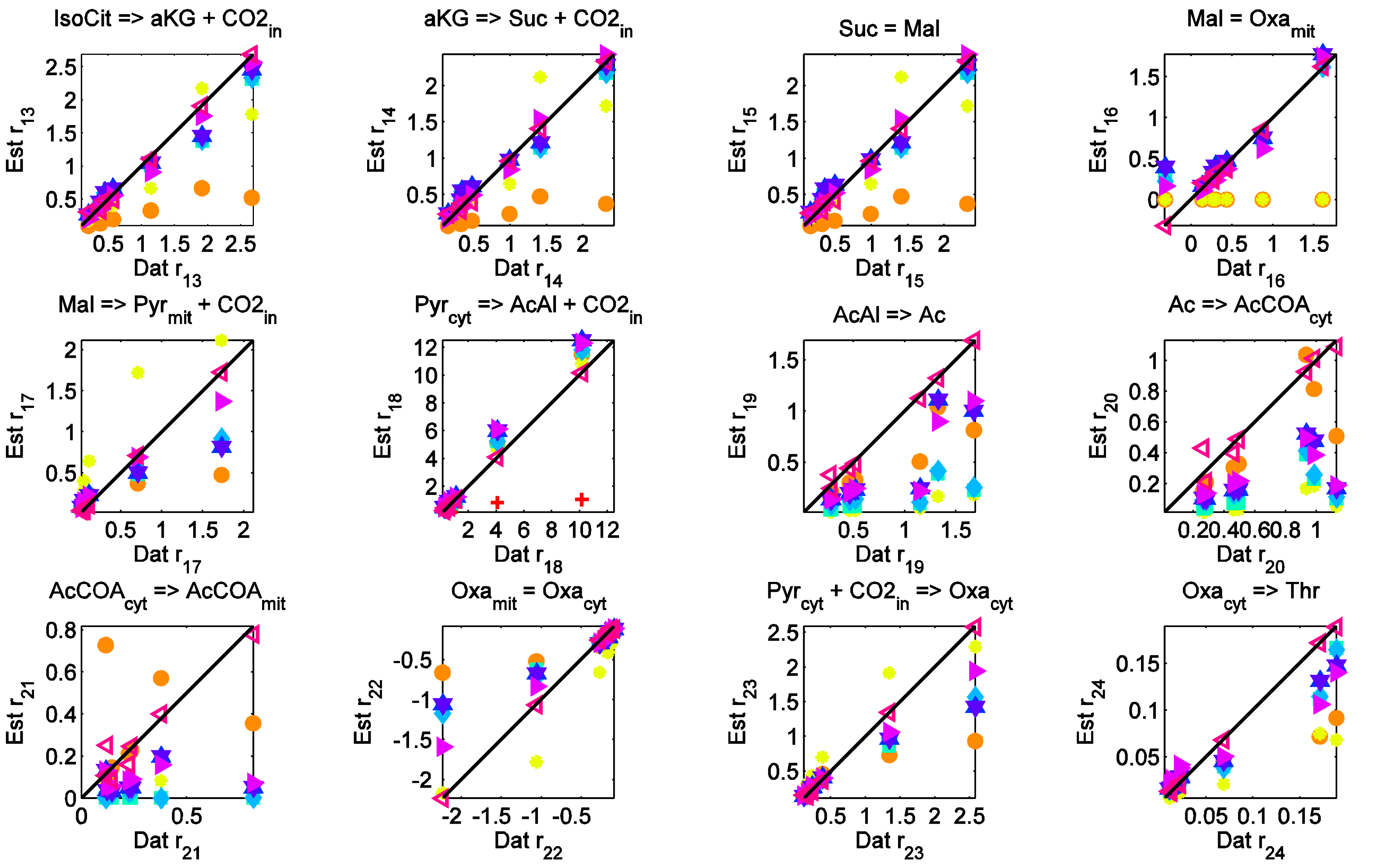

IsoCit => aKG + CO2in
Est r13
Dat r13
aKG => Suc + CO2in
Est r14
Dat r14
Suc = Mal
Est r15
Dat r15
Mal = Oxamit
Est r16
Dat r16
Mal => Pyrmit + CO2in
Est r17
Dat r17
Pyrcyt => AcAl + CO2in
Est r18
Dat r18
AcAl => Ac
Est r19
Dat r19
Ac => AcCOAcyt
Est r20
Dat r20
AcCOAcyt => AcCOAmit
Est r21
Dat r21
Oxamit = Oxacyt
Est r22
Dat r22
Pyrcyt + CO2in => Oxacyt
Est r23
Dat r23
Oxacyt => Thr
Est r24
Dat r24

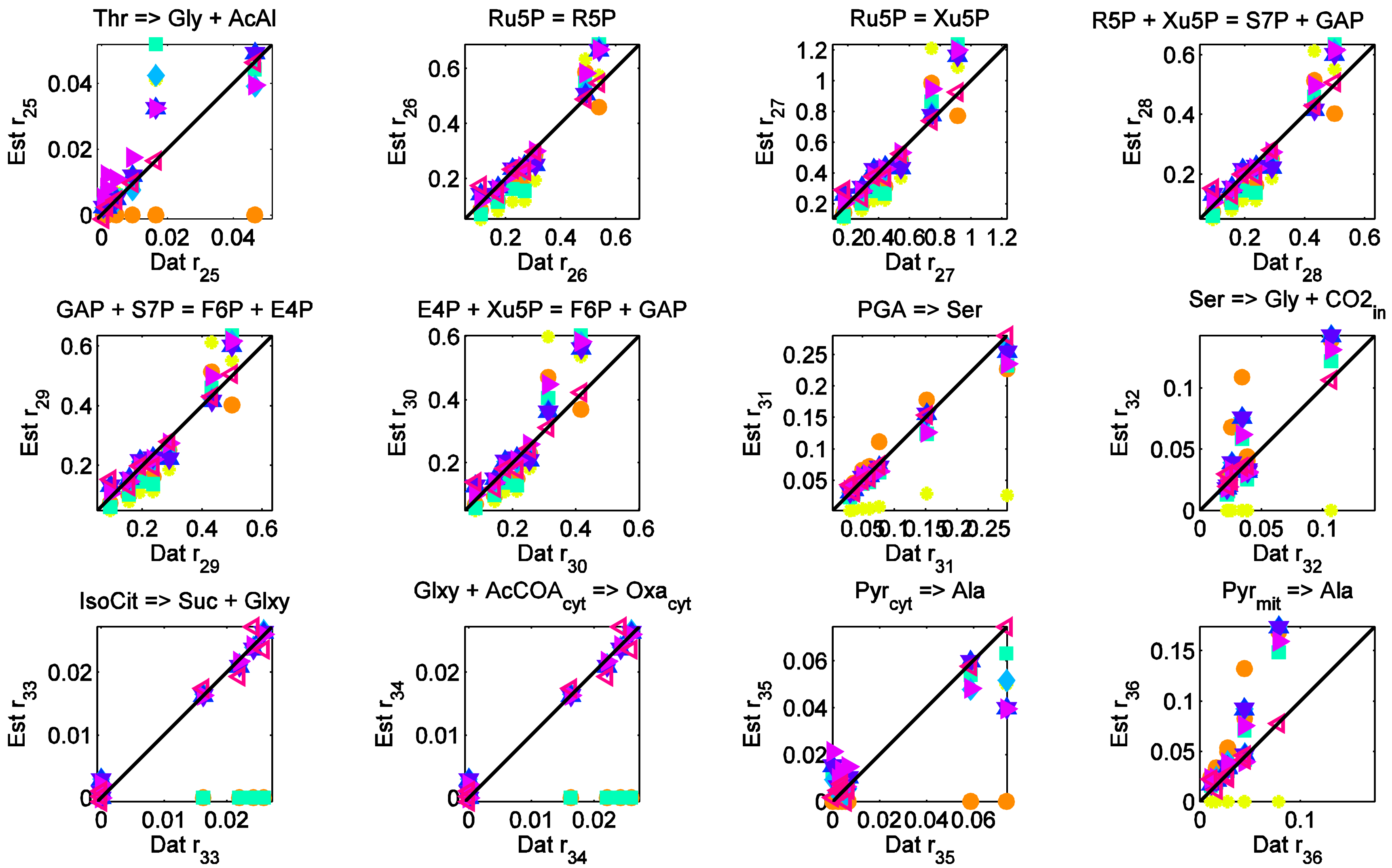

Thr => Gly + AcAl
Est r25
Dat r25
Ru5P = R5P
Est r26
Dat r26
Ru5P = Xu5P
Est r27
Dat r27
R5P + Xu5P = S7P + GAP
Est r28
Dat r28
GAP + S7P = F6P + E4P
Est r29
Dat r29
E4P + Xu5P = F6P + GAP
Est r30
Dat r30
PGA => Ser
Est r31
Dat r31
Ser => Gly + CO2in
Est r32
Dat r32
IsoCit => Suc + Glxy
Est r33
Dat r33
Glxy + AcCOAcyt => Oxacyt
Est r34
Dat r34
Pyrcyt => Ala
Est r35
Dat r35
Pyrmit => Ala
Est r36
Dat r36

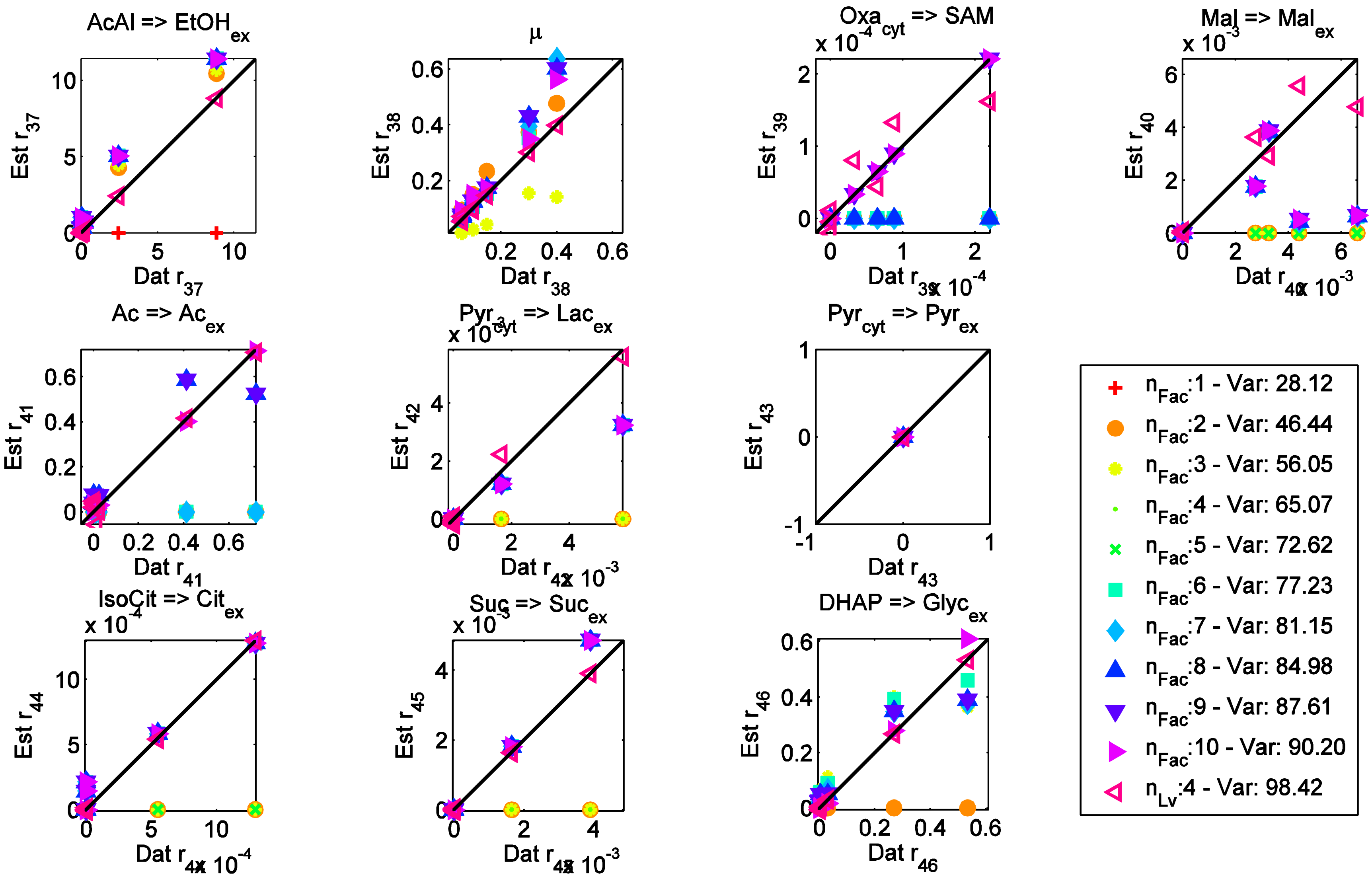

AcAl => EtOH_ex
Est r_37
Dat r_37
μ
Est r_38
Dat r_38
Oxa_cyt => SAM
x 10^-4
Est r_39
Dat r_39 x 10^-4
Mal => Mal_ex
x 10^-3
Est r_40
Dat r_40 x 10^-3
Ac => Ac_ex
Est r_41
Dat r_41
Pyr_cyt => Lac_ex
x 10^-3
Est r_42
Dat r_42 x 10^-3
Pyr_cyt => Pyr_ex
Est r_43
Dat r_43
IsoCit => Cit_ex
x 10^-4
Est r_44
Dat r_44 x 10^-4
Suc => Suc_ex
x 10^-3
Est r_45
Dat r_45 x 10^-3
DHAP => Glyc_ex
Est r_46
Dat r_46
n_Fac:1 - Var: 28.12
n_Fac:2 - Var: 46.44
n_Fac:3 - Var: 56.05
n_Fac:4 - Var: 65.07
n_Fac:5 - Var: 72.62
n_Fac:6 - Var: 77.23
n_Fac:7 - Var: 81.15
n_Fac:8 - Var: 84.98
n_Fac:9 - Var: 87.61
n_Fac:10 - Var: 90.20
n_Lv:4 - Var: 98.42

Active Elementary Modes

In the following ten pages, the active Elementary Flux Modes selected by PEMA with ten factors are highlighted in different colors in the metabolic network, which was adapted from Hayakawa et al [2].

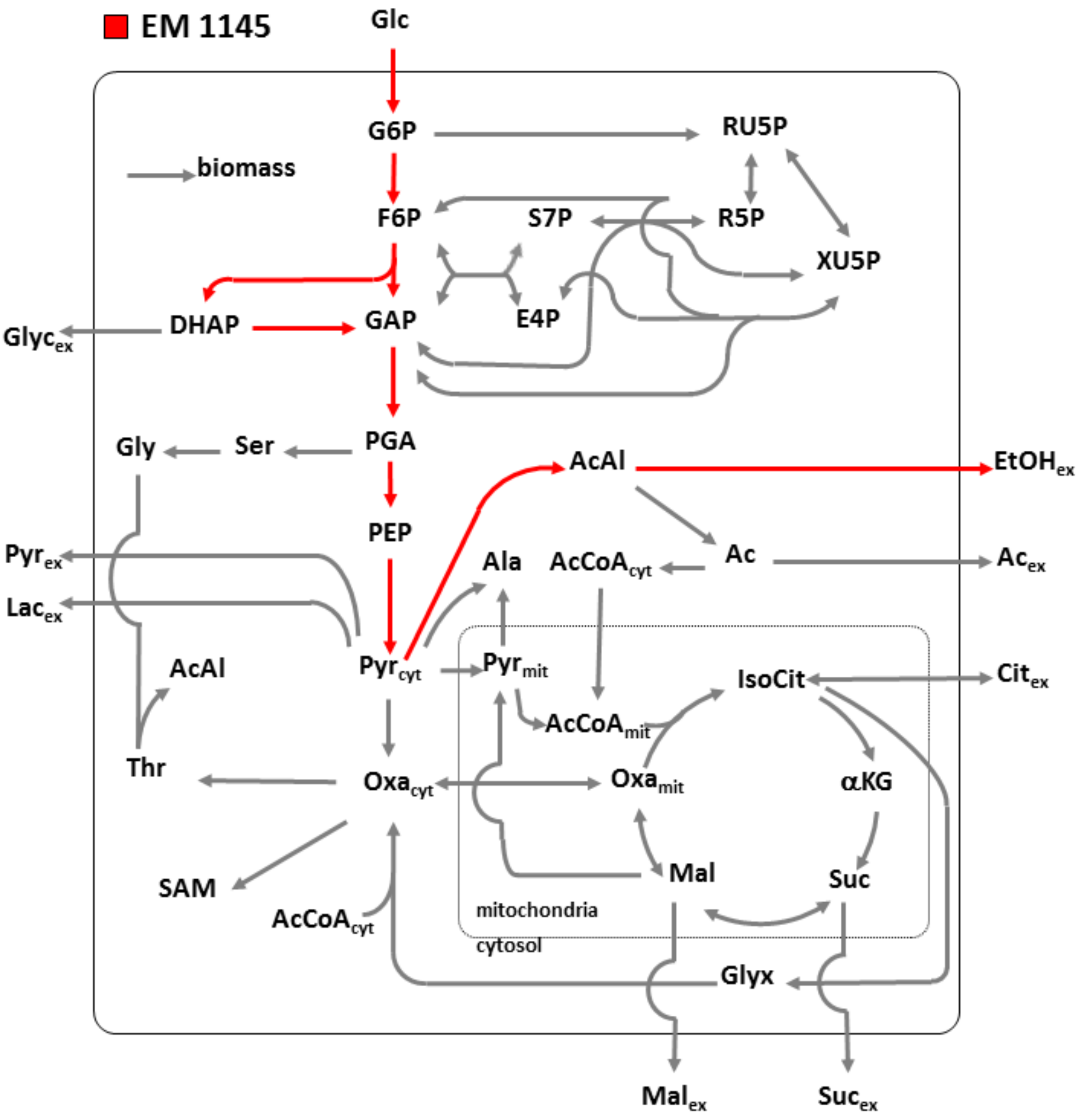

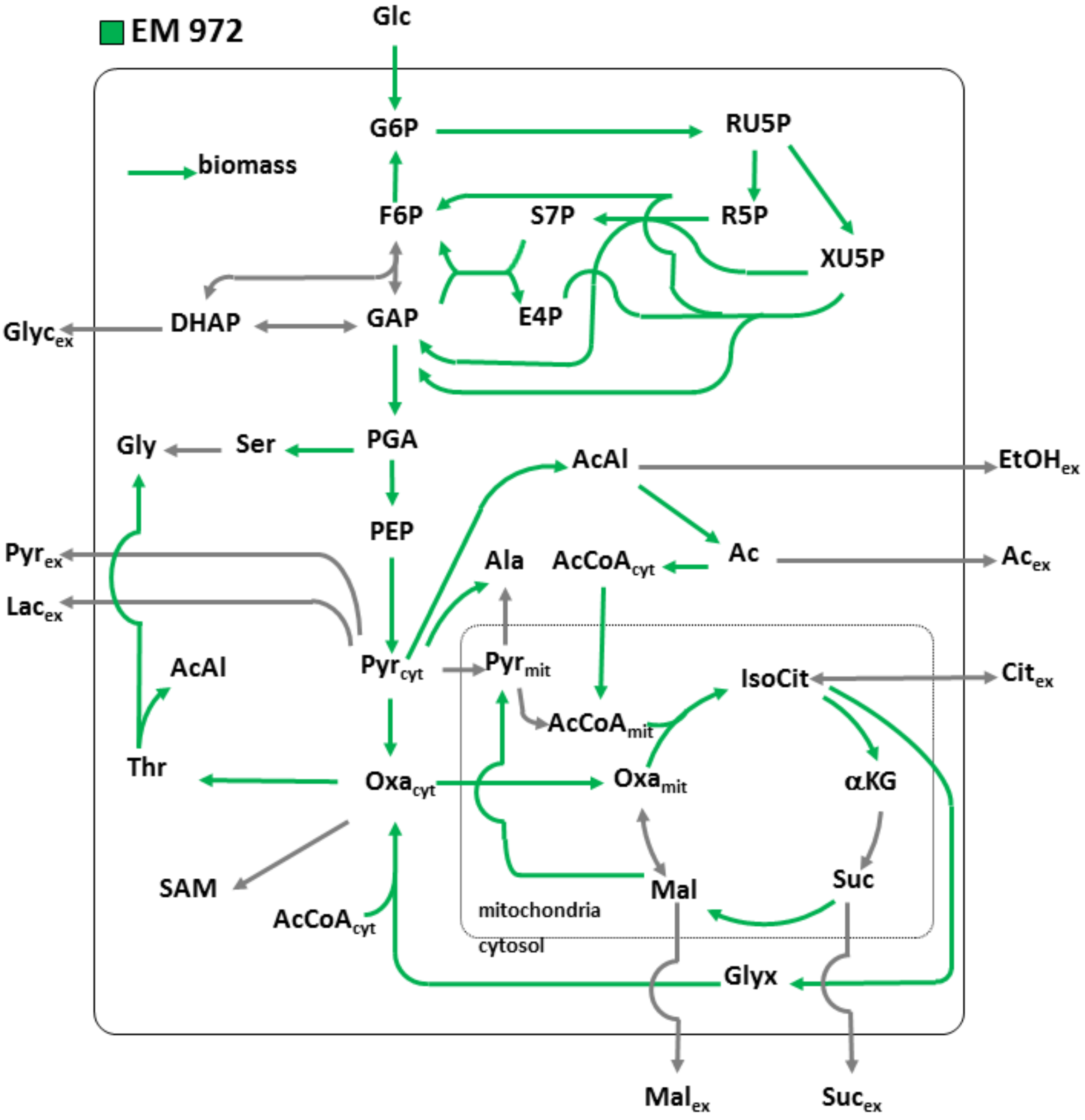

EM 972
Glc
G6P
RU5P
biomass
F6P
S7P
R5P
XU5P
Glyc$_{ex}$
DHAP
GAP
E4P
PGA
Gly
Ser
AcAl
EtOH$_{ex}$
PEP
Pyr$_{ex}$
Ala
AcCoA$_{cyt}$
Ac
Ac$_{ex}$
Lac$_{ex}$
AcAl
Pyr$_{cyt}$
Pyr$_{mit}$
IsoCit
Cit$_{ex}$
AcCoA$_{mit}$
Thr
Oxa$_{cyt}$
Oxa$_{mit}$
αKG
SAM
AcCoA$_{cyt}$
Mal
Suc
mitochondria
cytosol
Glyx
Mal$_{ex}$
Suc$_{ex}$

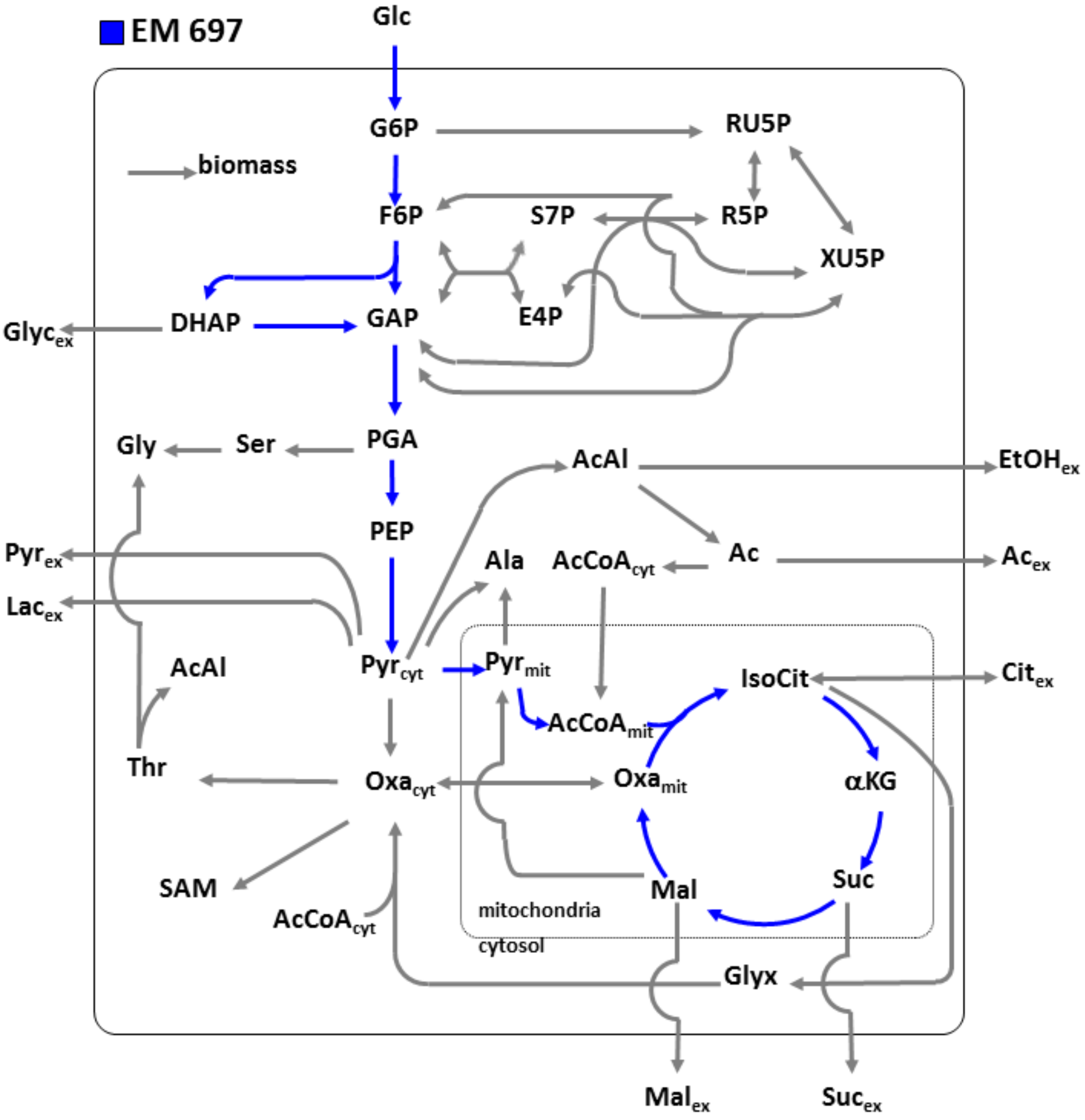
EM 697
Glc
G6P
RU5P
biomass
F6P
S7P
R5P
XU5P
DHAP
GAP
E4P
Glyc$_{ex}$
PGA
Ser
Gly
AcAl
EtOH$_{ex}$
PEP
Ac
Ac$_{ex}$
Pyr$_{ex}$
Ala
AcCoA$_{cyt}$
Lac$_{ex}$
AcAl
Pyr$_{cyt}$
Pyr$_{mit}$
IsoCit
Cit$_{ex}$
AcCoA$_{mit}$
Thr
Oxa$_{cyt}$
Oxa$_{mit}$
αKG
SAM
Mal
Suc
AcCoA$_{cyt}$
mitochondria
cytosol
Glyx
Mal$_{ex}$
Suc$_{ex}$

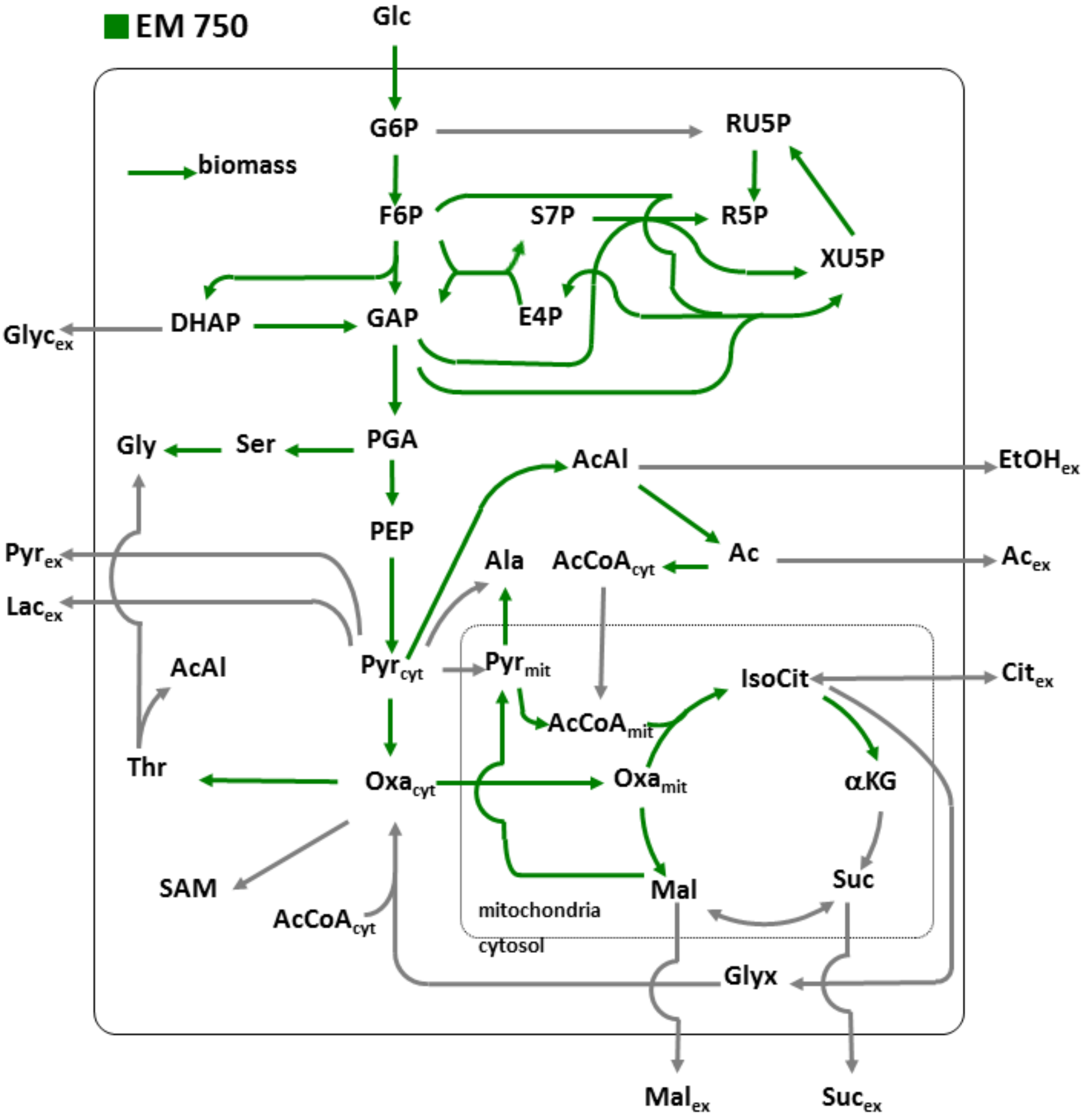

EM 750
Glc
G6P
RU5P
biomass
F6P
S7P
R5P
XU5P
Glyc$_{ex}$
DHAP
GAP
E4P
Gly
Ser
PGA
AcAl
EtOH$_{ex}$
PEP
Ac
Pyr$_{ex}$
Ala
AcCoA$_{cyt}$
Ac$_{ex}$
Lac$_{ex}$
AcAl
Pyr$_{cyt}$
Pyr$_{mit}$
IsoCit
Cit$_{ex}$
AcCoA$_{mit}$
Thr
Oxa$_{cyt}$
Oxa$_{mit}$
αKG
SAM
Mal
Suc
AcCoA$_{cyt}$
mitochondria
cytosol
Glyx
Mal$_{ex}$
Suc$_{ex}$

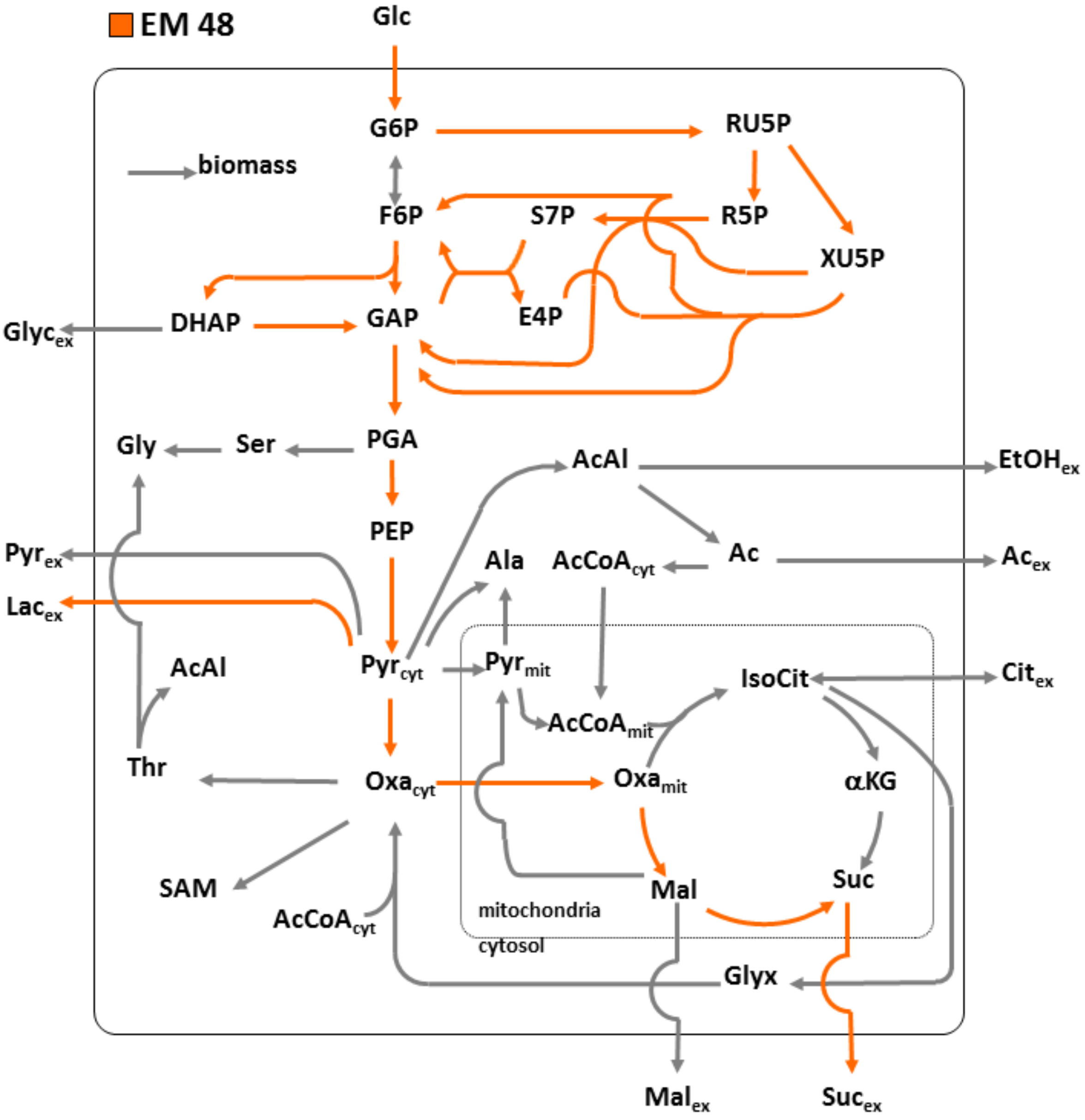
EM 48
Glc
G6P
RU5P
biomass
F6P
S7P
R5P
XU5P
DHAP
GAP
E4P
Glyc$_{ex}$
PGA
Gly
Ser
AcAl
EtOH$_{ex}$
PEP
Pyr$_{ex}$
Ala
AcCoA$_{cyt}$
Ac
Ac$_{ex}$
Lac$_{ex}$
AcAl
Pyr$_{cyt}$
Pyr$_{mit}$
IsoCit
Cit$_{ex}$
AcCoA$_{mit}$
Thr
Oxa$_{cyt}$
Oxa$_{mit}$
αKG
SAM
AcCoA$_{cyt}$
Mal
Suc
mitochondria
cytosol
Glyx
Mal$_{ex}$
Suc$_{ex}$

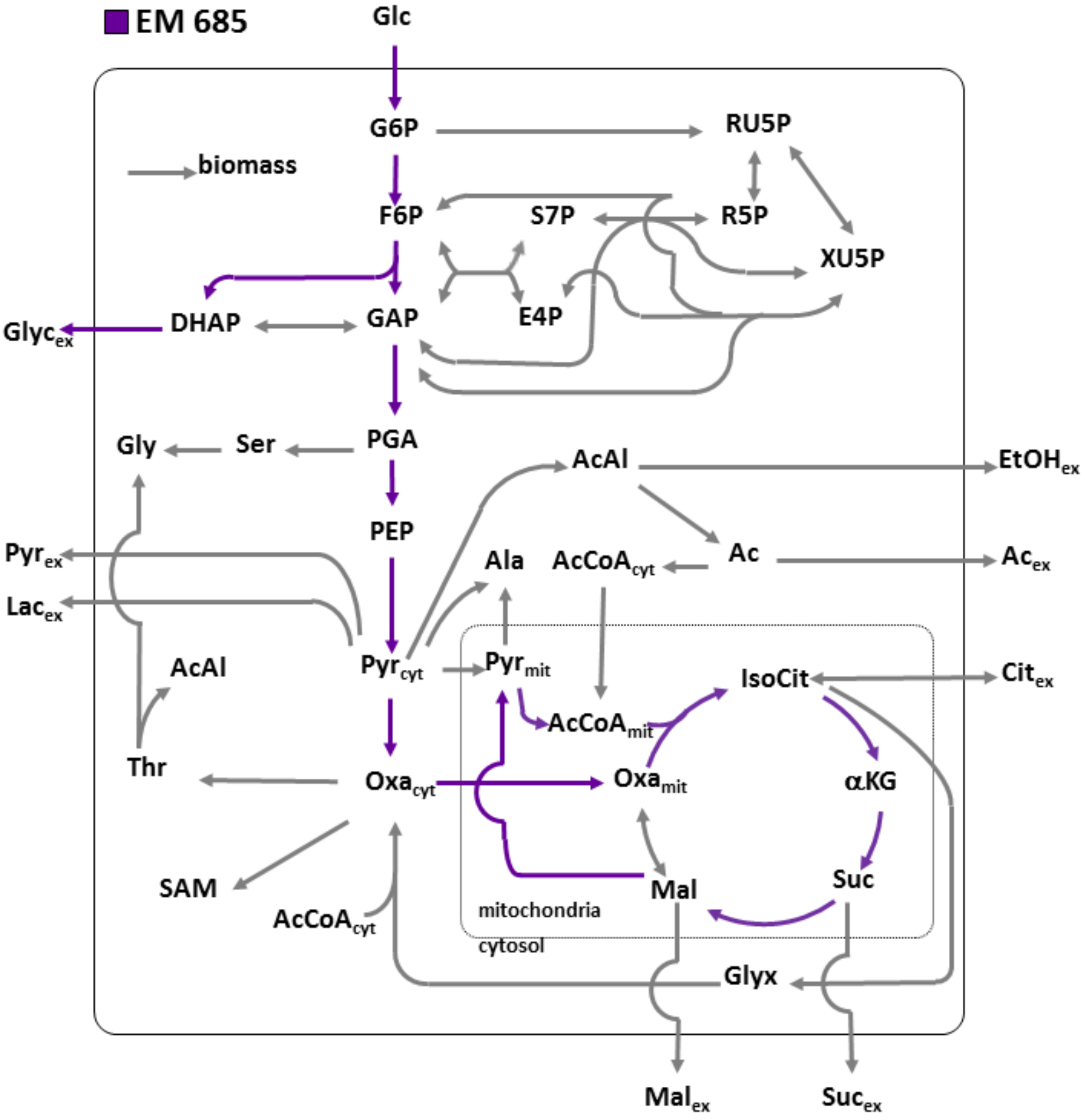
EM 685
Glc
G6P
RU5P
biomass
F6P
S7P
R5P
XU5P
DHAP
GAP
E4P
Glyc$_{ex}$
PGA
Gly
Ser
AcAl
EtOH$_{ex}$
PEP
Ac
Ac$_{ex}$
Pyr$_{ex}$
Ala
AcCoA$_{cyt}$
Lac$_{ex}$
AcAl
Pyr$_{cyt}$
Pyr$_{mit}$
IsoCit
Cit$_{ex}$
AcCoA$_{mit}$
Thr
Oxa$_{cyt}$
Oxa$_{mit}$
αKG
SAM
AcCoA$_{cyt}$
Mal
Suc
mitochondria
cytosol
Glyx
Mal$_{ex}$
Suc$_{ex}$

EM 31

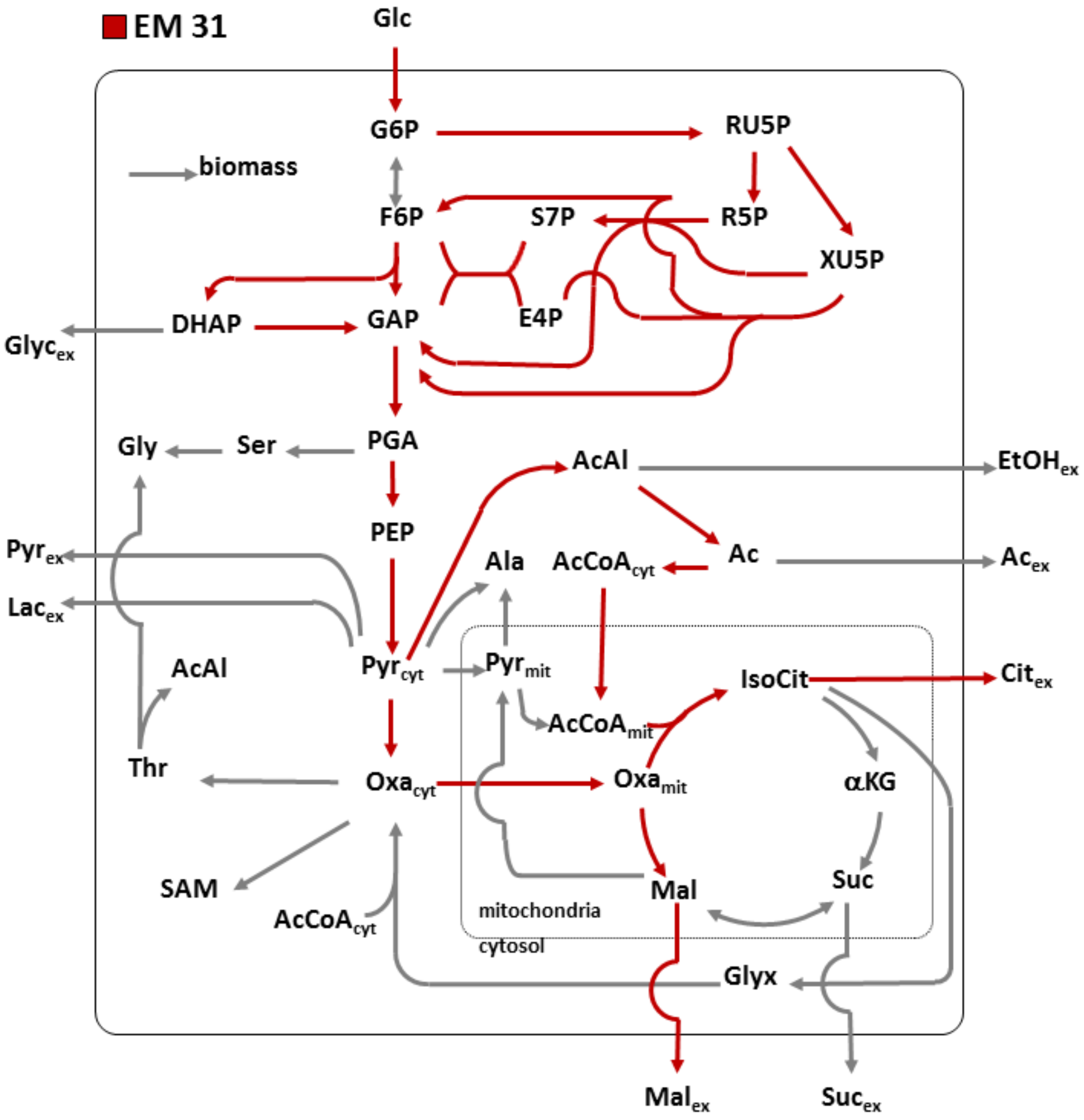

Glc
G6P
RU5P
biomass
F6P
S7P
R5P
XU5P
DHAP
GAP
E4P
Glyc$_{ex}$
PGA
Gly
Ser
AcAl
EtOH$_{ex}$
PEP
Ac
Pyr$_{ex}$
Ala
AcCoA$_{cyt}$
Ac$_{ex}$
Lac$_{ex}$
AcAl
Pyr$_{cyt}$
Pyr$_{mit}$
IsoCit
Cit$_{ex}$
AcCoA$_{mit}$
Thr
Oxa$_{cyt}$
Oxa$_{mit}$
αKG
SAM
AcCoA$_{cyt}$
Mal
Suc
mitochondria
cytosol
Glyx
Mal$_{ex}$
Suc$_{ex}$

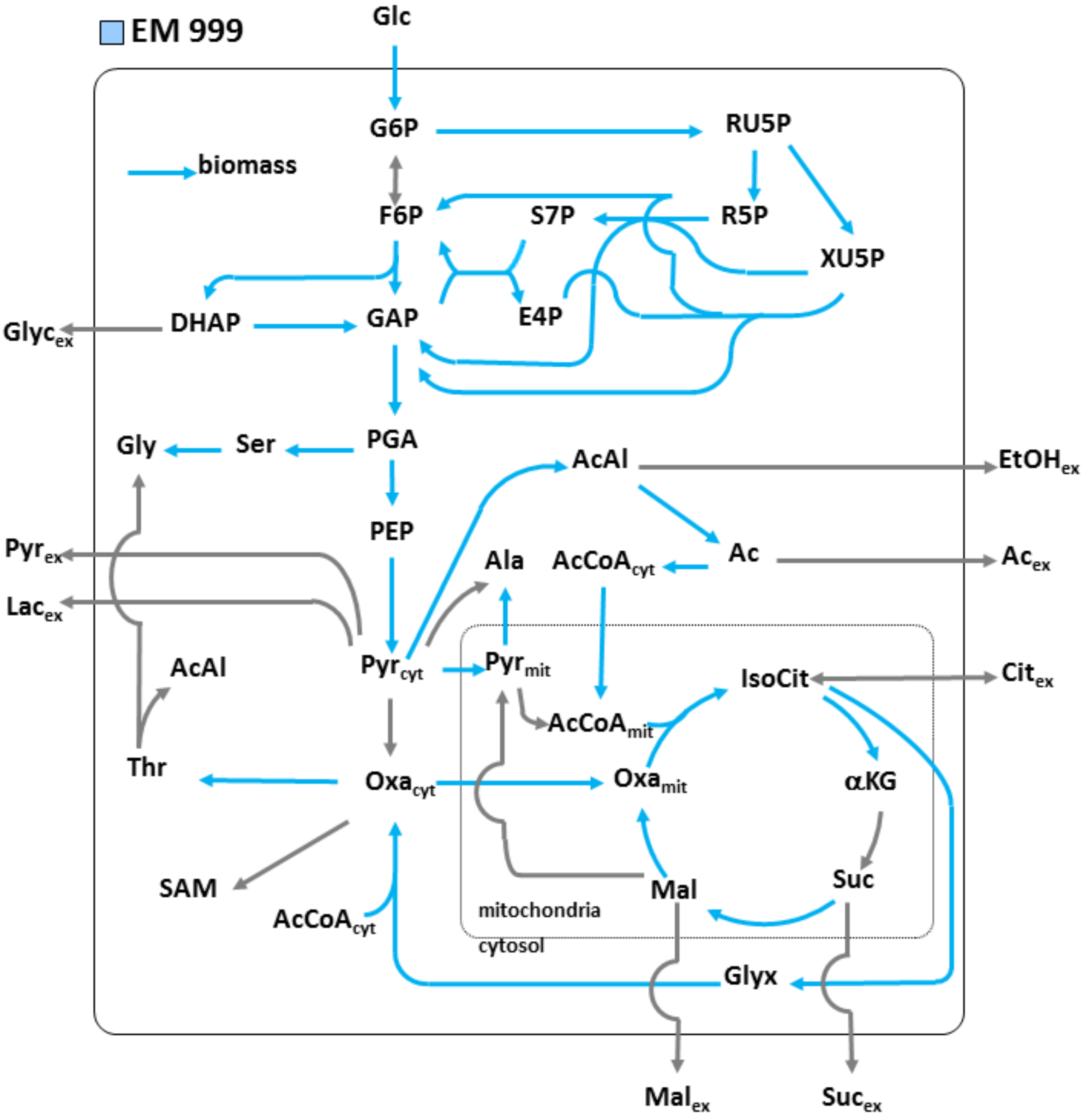
EM 999
Glc
G6P
RU5P
biomass
F6P
S7P
R5P
XU5P
Glyc$_{ex}$
DHAP
GAP
E4P
Gly
Ser
PGA
AcAl
EtOH$_{ex}$
PEP
Pyr$_{ex}$
Ala
AcCoA$_{cyt}$
Ac
Ac$_{ex}$
Lac$_{ex}$
AcAl
Pyr$_{cyt}$
Pyr$_{mit}$
IsoCit
Cit$_{ex}$
AcCoA$_{mit}$
Thr
Oxa$_{cyt}$
Oxa$_{mit}$
αKG
SAM
AcCoA$_{cyt}$
Mal
Suc
mitochondria
cytosol
Glyx
Mal$_{ex}$
Suc$_{ex}$

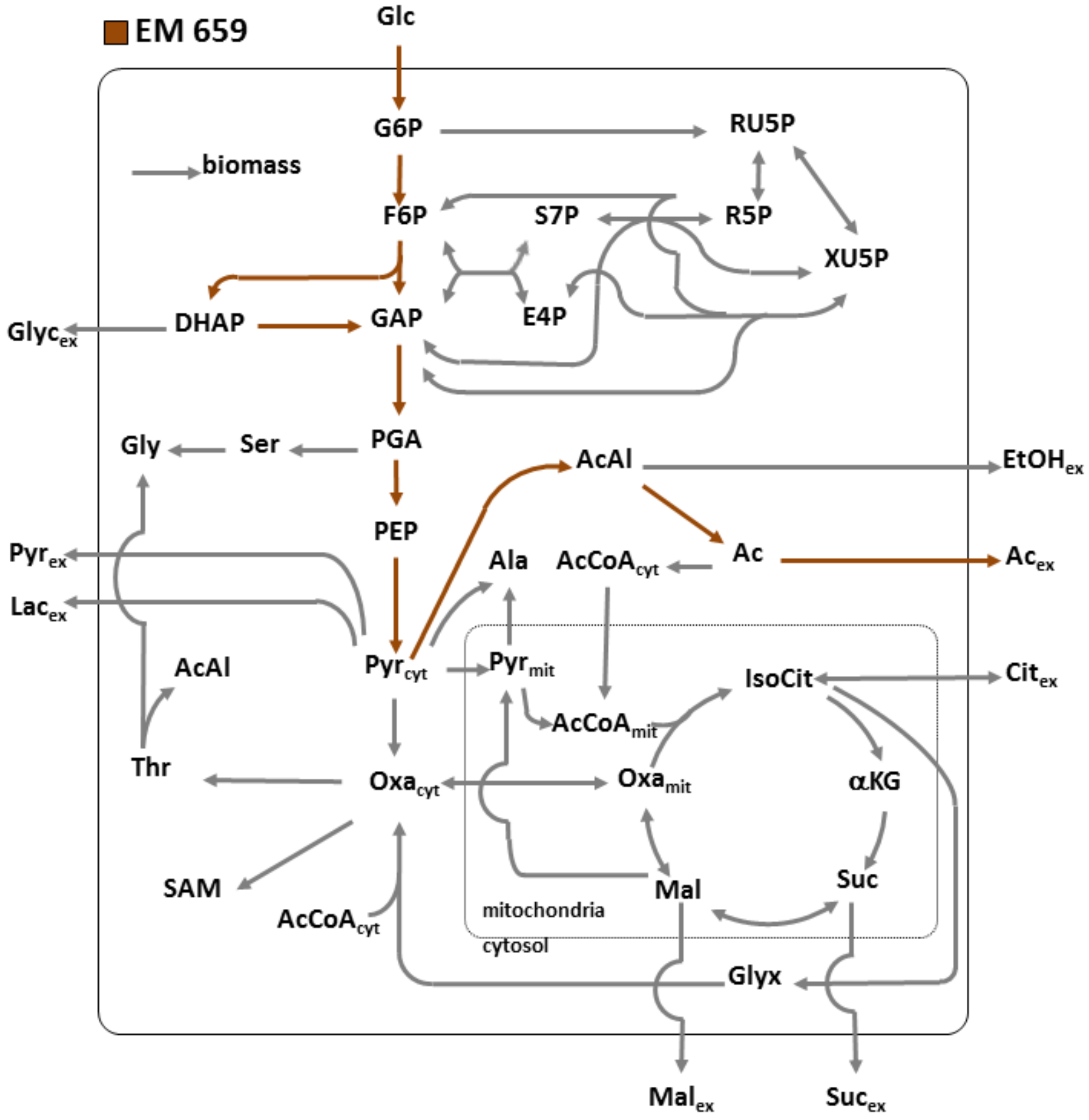
EM 659
Glc
G6P
RU5P
biomass
F6P
S7P
R5P
XU5P
DHAP
GAP
E4P
$Glyc_{ex}$
PGA
Gly
Ser
AcAl
$EtOH_{ex}$
PEP
Ala
$AcCoA_{cyt}$
Ac
Ac_{ex}
Pyr_{ex}
Lac_{ex}
AcAl
Pyr_{cyt}
Pyr_{mit}
IsoCit
Cit_{ex}
$AcCoA_{mit}$
Thr
Oxa_{cyt}
Oxa_{mit}
αKG
SAM
$AcCoA_{cyt}$
Mal
Suc
mitochondria
cytosol
Glyx
Mal_{ex}
Suc_{ex}

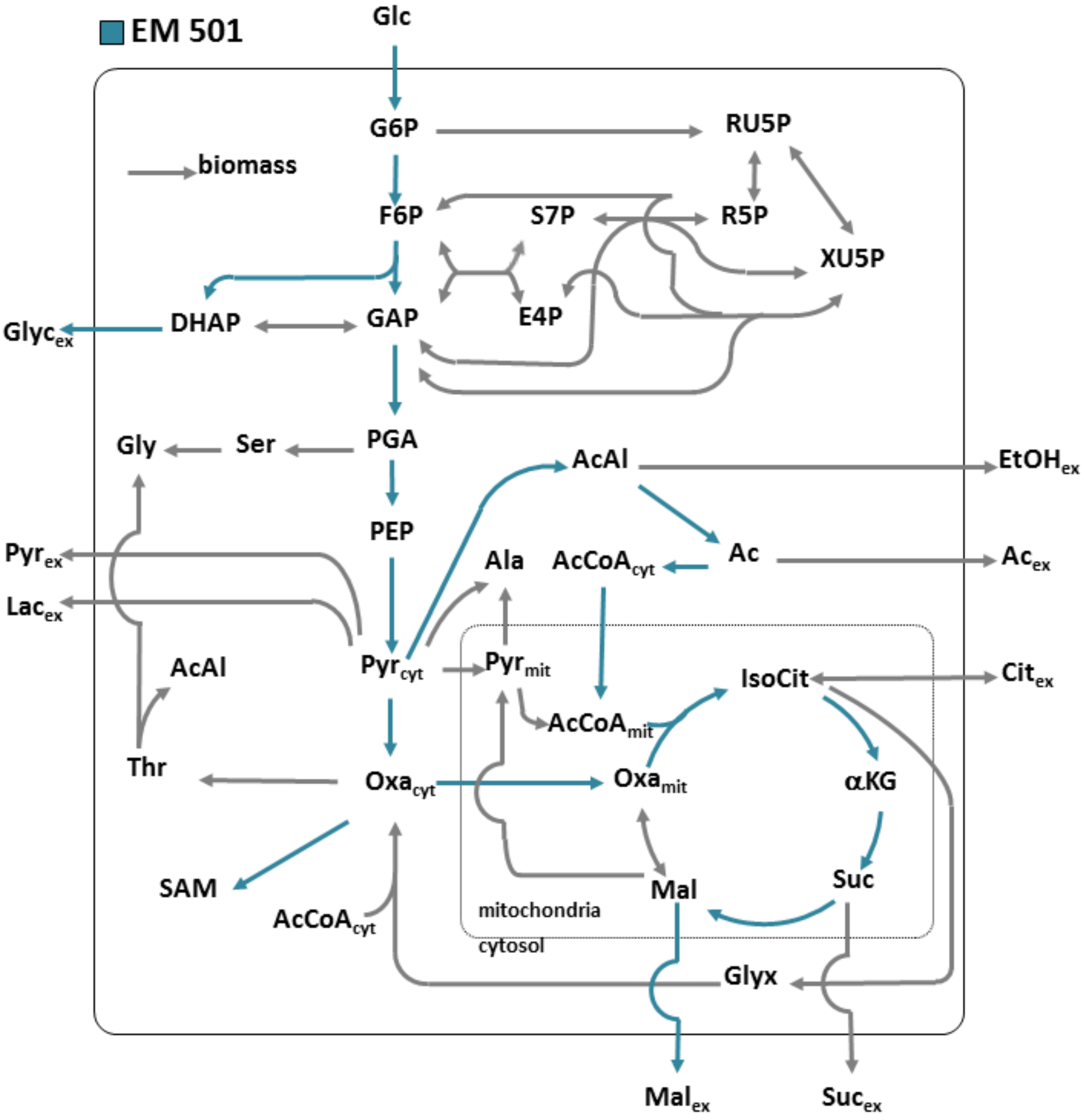
EM 501
Glc
G6P
RU5P
biomass
F6P
S7P
R5P
XU5P
Glyc$_{ex}$
DHAP
GAP
E4P
PGA
Gly
Ser
AcAl
EtOH$_{ex}$
PEP
Ac
Ac$_{ex}$
Pyr$_{ex}$
Ala
AcCoA$_{cyt}$
Lac$_{ex}$
AcAl
Pyr$_{cyt}$
Pyr$_{mit}$
IsoCit
Cit$_{ex}$
AcCoA$_{mit}$
Thr
Oxa$_{cyt}$
Oxa$_{mit}$
αKG
SAM
AcCoA$_{cyt}$
Mal
Suc
mitochondria
cytosol
Glyx
Mal$_{ex}$
Suc$_{ex}$

References

1. Tortajada M, Llaneras F, Pico J (2010) Validation of a constraint-based model of Pichia pastoris metabolism under data scarcity. BMC Systems Biology 4: 115.
2. Hayakawa K, Kajihata S, Matsuda F, Shimizu H 13C-metabolic flux analysis in S-adenosyl-l-methionine production by Saccharomyces cerevisiae. Journal of Bioscience and Bioengineering.
3. Gianchandani EP, Oberhardt MA, Burgard AP, Maranas CD, Papin JA (2008) Predicting biological system objectives de novo from internal state measurements. BMC Bioinformatics 9: 43-43.
